# Reflection from a multi-species material and its transmitted effective wavenumber

**DOI:** 10.1098/rspa.2017.0864

**Published:** 2018-04-11

**Authors:** Artur L. Gower, Michael J. A. Smith, William J. Parnell, I. David Abrahams

**Affiliations:** 1 School of Mathematics, University of Manchester, Oxford Road, Manchester M13 9PL, UK; 2 Isaac Newton Institute for Mathematical Sciences, 20 Clarkson Road, Cambridge CB3 0EH, UK

**Keywords:** multiple scattering, polydisperse, ensemble average, random media, size distribution, homogenization

## Abstract

We formally deduce closed-form expressions for the transmitted effective wavenumber of a material comprising multiple types of inclusions or particles (multi-species), dispersed in a uniform background medium. The expressions, derived here for the first time, are valid for moderate volume fractions and without restriction on the frequency. We show that the multi-species effective wavenumber is not a straightforward extension of expressions for a single species. Comparisons are drawn with state-of-the-art models in acoustics by presenting numerical results for a concrete and a water–oil emulsion in two dimensions. The limit of when one species is much smaller than the other is also discussed and we determine the background medium felt by the larger species in this limit. Surprisingly, we show that the answer is not the intuitive result predicted by self-consistent multiple scattering theories. The derivation presented here applies to the scalar wave equation with cylindrical or spherical inclusions, with any distribution of sizes, densities and wave speeds. The reflection coefficient associated with a halfspace of multi-species cylindrical inclusions is also formally derived.

## 1. Summary

Materials comprising mixtures of diverse particles, inclusions, defects or inhomogeneities dispersed inside a background medium arise in a wide range of applications, including composite materials, emulsions, gases, polymers, foods and paints. We will refer to these as multi-species materials. Of great importance is the ability to characterize these materials and their *microstructure*, such as particle size distribution and volume fractions. One approach to do this is to employ waves, including electromagnetic, acoustic and elastodynamic waves. If either the receivers are much larger than the inclusions, or the wavelength is much longer than the inclusions, then the receivers will measure the *ensemble-averaged* properties of the wave [1]. This includes the wave speed, attenuation and reflection. Even methods that estimate fluctuations of the wave on smaller scales, such as the averaged intensity, often require the ensemble-averaged wave properties as a first step [2–4]. So in order to improve material characterization, or to design materials with tailored properties, a crucial step is to rigorously calculate the sound speed and attenuation for multi-species materials.

In this paper, we present and formally deduce the effective wavenumber and reflected field of a plane wave scattered by a material comprising different families, or *species*, of particles with distributions of sizes and properties. The work here differs from the existing literature as our results are not limited to low frequencies and are valid for moderate number density. This is achieved by extending the methods introduced in [5] for calculating the effective transmission into a halfspace of a single-species material.

Our approach does not rely on an extinction theorem or the manipulation of divergent integrals or series. The one assumption that is employed is the *quasi-crystalline approximation* [6]. For a single species, this approximation is supported by theoretical [7,8], numerical [9] and experimental [10] evidence; however, the authors are unaware of any rigorous bounds for the error introduced by this approximation. We remark that the quasi-crystalline approximation (QCA) makes no assumption on the material properties, so in principal it is consistent for weak scattering, low or high frequency, or in dense or sparse mixtures. It is also exact when there is only one possible configuration for the particles, for example when the particle centres lie on the coordinates of a crystal lattice (Lax [11], where QCA is discussed under (4.3)). For simplicity, we also restrict attention to the case of circular cylindrical or spherical particles, although our methods can be extended to the case of general-shaped particles by using Waterman’s T-matrix approach, e.g. [12–14].

In the context of electromagnetic wave scattering, methods for predicting wave propagation and reflection for multi-species material have previously been developed [3,4,15]. These models have been useful for interpreting data from remote sensing, although it appears that such models cannot systematically reproduce experimental results [16]. In numerous contexts, but particularly in the context of electromagnetics, the standard approach is to employ the Lippman–Schwinger formulation [17,18]. However, such a formulation is restrictive as it is not valid for magnetic media in the electromagnetism context or for scatterers with varying density in acoustics, as identified in [19]. Although it is possible to extend the Lippman–Schwinger formulation to account for this effect [19], we found it simpler to extend the multiple scattering theory [2,20,21].

Our approach is also in contrast to coupled-phase theory where the first step is to estimate the ensemble average of the governing equations [22], without explicitly considering multiple scattering. Although this method can accommodate hydrodynamic interactions and has been extended to polydisperse inclusions (multi-species) it does not completely capture multiple scattering [23,24].

A suggestion for calculating the multi-species effective wavenumber came from Waterman & Truell, eqn (3.25a) in the conclusion of [25]. Their suggested formula has been extensively employed in acoustics, e.g. [26–28]. However, their formula is only valid for low frequency and dilute distributions of particles [21], so it does not properly account for multiple scattering. The approach in [25] combined with [29] led eventually to the state-of-the-art models for the effective acoustic wavenumber in colloidal dispersions [28]. We numerically compare our results with these authors.

Given an overall particle number density n and background wavenumber *k*, our main results for a multi-species material comprising *circular cylinders* are the effective transmitted wavenumber:

1.1 $$k_{*}^{2}=k^{2}-4in\;\langle \;f_{\circ }\rangle (0)-4in^{2}\langle \;f_{\circ \circ }\rangle (0)+O(n^{3})$$

and for an incident wave *u*_in_=*e*^i*αx*+i*βy*^, with $\left(\alpha ,\beta )=k(\cos\theta_{\mathrm{in}},\sin\theta_{\mathrm{in}}\right)$, the averaged reflected wave from the inhomogeneous halfspace,

1.2 $$\langle u_{\mathrm{ref}}\rangle =\frac{n}{\alpha^{2}}[R_{1}+nR_{2}]\;e^{-i\alpha x+i\beta y}+O(n^{3}),$$

where

1.3 $$\begin{array}{rl} R_{1} & \;=i\langle \;f_{\circ }\rangle (\theta_{\mathrm{ref}}),\;\theta_{\mathrm{ref}}=\pi -2\theta_{\mathrm{in}}, \end{array}$$

1.4 $$\begin{array}{rl} R_{2} & \;=\frac{2\langle \;f_{\circ }\rangle (0)}{\alpha^{2}}\left[\frac{\alpha \beta }{k^{2}}{\left\langle \;f_{\circ }\right\rangle }^{\prime }(\theta_{\mathrm{ref}})-\langle \;f_{\circ }\rangle (\theta_{\mathrm{ref}})\right]+i\langle \;f_{\circ \circ }\rangle (\theta_{\mathrm{ref}}) \end{array}$$

and the functions 〈 *f*_°_〉 and 〈 *f*_°°_〉 are defined in (4.12) and (4.13). The formula (1.2) is briefly deduced in §7a, and in figure 7 we give a pictorial representation, although we stress that the choice *θ*_ref_=*π*−2*θ*_in_ is not due to a simple geometric argument, but appears from rigorous derivations. From the reflection coefficient (1.2), it is possible to choose effective material properties [30]. However, because the reflection coefficient depends on the angle of incidence via 〈 *f*_°_〉(*θ*_ref_) and 〈 *f*_°°_〉(*θ*_ref_), it is likely that these effective material properties change with the angle of incidence.

In the electronic supplementary material, we provide a brief self-contained version of these formulae, and the corresponding result for spherical particles, both for a finite number of species. We also provide open source code that implements these formulae [31]. For spherical inclusions, the effective transmitted acoustic wavenumber becomes

1.5 $$k_{*}^{2}=k^{2}-n\frac{4\pi i}{k}\langle F_{\circ }\rangle (0)+n^{2}\frac{{\left(4\pi \right)}^{2}}{k^{4}}\langle F_{\circ \circ }\rangle +O(n^{3}),$$

where 〈*F*_°_〉 and 〈*F*_°°_〉 are functions associated with scattering from the spherical particle and are defined in (A.2). Note that 〈*F*_°°_〉 has no *θ* dependency. For a longer discussion of multiple scattering from spheres, see [30].

By developing *multi-species* formulae valid for higher number densities and frequencies, we open up the possibility of characterizing and designing a wide range of advanced materials. The effects of multiple scattering appear only for moderate number density, i.e. in the term 〈 *f*_°°_〉(0) in (1.1) and 〈*F*_°°_〉 in (1.5). One important consequence of this multiple scattering term is that a multi-species material can exhibit properties not exhibited by that of the background medium with only one constituent species. We stress that even for just two types of circular cylindrical particles, the effects of multiple scattering are neither intuitive nor easily deduced from the single-species case. This becomes apparent in the simple example of a multi-species material where one species is much smaller than the other. In this scenario, we compare our expression for the multi-species effective wavenumber with the state-of-the-art models from acoustics [28] and a *self-consistent* type approximation [32–34], which can be calculated from the single-species formula via an iterative approach: first one *homogenizes* the small particle and background mixture before considering the multiple scattering of the larger particles in the new (homogenized) background medium. We show analytically that this naive self-consistent methodology is not even correct in the low-frequency limit. This is then demonstrated numerically for the cases of an emulsion and concrete. Our results are in in-line with [35], who discuss an effective medium model of a three-phased material in the low-frequency limit.

The outline of this paper is as follows. In §2, we describe the exact theory of multiple scattering for *N* cylinders of any radius, density and sound speed. From there we calculate the effective (ensemble-averaged) equations and apply statistical approximations in §3. In §4, we deduce the governing system for the effective wavenumbers at arbitrary total number density and arbitrary frequency, before specializing the result to the case of moderate number fraction and low frequency. In §5, we investigate the specific, representative case of two types of circular cylindrical species and compare different approximations graphically. To calculate the reflected or transmitted wave we also need the effective amplitude, which we calculate in §7 followed by the effective reflected wave. We close in §8, where we discuss avenues for improvement of the techniques and more general further work.

## 2. Multipole method for cylinders

In this section, we describe the exact theory for scalar multiple wave scattering from a finite number *N* of circular cylinders possessing different densities, wave speeds and radii. Parameters associated with the medium are summarized in table 1. Naturally, the system of equations describing this problem bears strong similarities to that obtained by Záviška (see references in [36] and in [5] for the single species circular cylindrical particle context). Assuming time-harmonic dependence of the form *e*^−i*ωt*^, the pressure *u* outside all the cylinders satisfies the scalar Helmholtz equation

2.1a $$\nabla^{2}u+k^{2}u=0$$

and inside the *j*th cylinder the pressure *u*_*j*_ satisfies

2.1b $$\nabla^{2}u_{j}+k_{j}^{2}u_{j}=0,\;\text{for}\;j=1,2,\ldots ,N,$$

where ∇^2^ is the two-dimensional Laplacian and

2.2 $$k=\frac{\omega }{c}\;\text{and}\;k_{j}=\frac{\omega }{c_{j}}.$$

We consider an incident plane wave

$$u_{\mathrm{in}}(x,y)=e^{i(\alpha x+\beta y)},\;\text{with}\;(\alpha ,\beta )=k(\cos\theta_{\mathrm{in}},\sin\theta_{\mathrm{in}})$$

and use for each cylinder the polar coordinates

**Figure 1.**
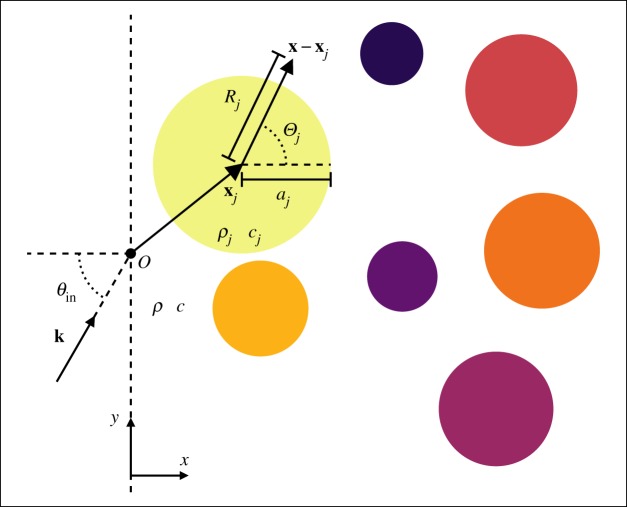
Represents a multi-species material comprising different species of cylinders to the right of the origin *O*=(0,0). The vector **x**_*j*_ points to the centre of the *j*th cylinder, with a local polar coordinate system (*R*_*j*_,*Θ*_*j*_). Each cylinder has a radius *a*_*j*_, density *ρ*_*j*_ and wave speed *c*_*j*_, while the background has density *ρ* and wave speed *c*. The vector **k** is the direction of the incident plane wave. (Online version in colour.)

2.3 $$R_{j}=∥\text{x}-{\text{x}}_{j}∥,\;\Theta_{j}=\arctan\left(\frac{y-y_{j}}{x-x_{j}}\right),$$

where **x**_*j*_ is the centre of the *j*th cylinder and **x**=(*x*,*y*) is an arbitrary point with origin *O*. (See figure 1 for a schematic of the material properties and coordinate systems.) Then we can define *u*_*j*_ as the scattered pressure field from the *j*th cylinder,

2.4 $$u_{j}(R_{j},\Theta_{j})=\sum_{m=-\infty }^{\infty }A_{j}^{m}Z_{j}^{m}H_{m}(kR_{j})\;e^{im\Theta_{j}},\;\text{for}\;R_{j}>a_{j},$$

where *H*_*m*_ are Hankel functions of the first kind, $A_{j}^{m}$ are arbitrary coefficients and $Z_{j}^{m}$ characterizes the type of scatterer:

2.5 $$Z_{j}^{m}=\frac{q_{j}J_{m}^{\prime }(ka_{j})J_{m}(k_{j}a_{j})-J_{m}(ka_{j})J_{m}^{\prime }(k_{j}a_{j})}{q_{j}H_{m}^{\prime }(ka_{j})J_{m}(k_{j}a_{j})-H_{m}(ka_{j})J_{m}^{\prime }(k_{j}a_{j})}=Z_{j}^{-m},$$

with *q*_*j*_=(*ρ*_*j*_*c*_*j*_)/(*ρc*). In the limits *q*_*j*_→0 or $q_{j}\to \infty$, the coefficients for Dirichlet or Neumann boundary conditions are recovered, respectively.

**Table 1.** Summary of material properties and notation. The index *j* refers to properties of the *j*th species, see figure 1 for an illustration.

background properties:		density *ρ*	sound speed *c*	
species properties:	number density $n_{j}$	density *ρ*_*j*_	sound speed *c*_*j*_	radius *a*_*j*_

The pressure outside all cylinders is the sum of the incident wave *u*_in_ and all scattered waves,

2.6 $$u(x,y)=u_{\mathrm{in}}(x,y)+\sum_{j=1}^{N}u_{j}(R_{j},\Theta_{j})$$

and the total field inside the *j*th cylinder is

2.7 $$u_{j}^{I}(R_{j},\Theta_{j})=\sum_{m=-\infty }^{\infty }B_{j}^{m}J_{m}(k_{j}R_{j})\;e^{im\Theta_{j}},\;\text{for}\;R_{j}<a_{j}.$$

In the above, *J*_*m*_ are Bessel functions of the first kind. The arbitrary constants $A_{j}^{m}$ and $B_{j}^{m}$ in (2.4) and (2.7) will be determined from the boundary conditions of the *j*th cylinder *R*_*j*_=*a*_*j*_. The boundary conditions of continuity of pressure and normal velocity on the cylinder boundaries are given, respectively, by

2.8 $$u=u_{j}^{I}\;\text{and}\;\frac{1}{\rho }\frac{\partial u}{\partial R_{j}}=\frac{1}{\rho_{j}}\frac{\partial u_{j}^{I}}{\partial R_{j}},\;\text{on}\;R_{j}=a_{j}\;\text{for}\;j=1,\ldots ,N,$$

recalling that *ρ* and *ρ*_*j*_ denote the material densities of the background and of the *j*th cylinder respectively. To impose the boundary conditions, we now express the relevant fields in terms of the (*R*_*j*_,*Θ*_*j*_) coordinate system. For the incident wave

2.9 $$u_{\mathrm{in}}(x,y)=I_{j}\;e^{ikr_{j}\cos(\theta_{j}-\theta_{\mathrm{in}})}=I_{j}\sum_{n=-\infty }^{\infty }\;e^{in(\pi /2-\Theta_{j}+\theta_{\mathrm{in}})}J_{n}(kR_{j}),$$

where *I*_*j*_=*u*_in_(*x*_*j*_,*y*_*j*_) following the Jacobi–Anger expansion [37]. For the scattered waves (2.4), we use Graf’s addition theorem (9.1.79) in [38],

2.10 $$H_{n}(kR_{\ell })\;e^{in\Theta_{\ell }}=\sum_{m=-\infty }^{\infty }H_{n-m}(kR_{\ell j})\;e^{i(n-m)\Theta_{\ell j}}J_{m}(kR_{j})\;e^{im\Theta_{j}},\;\text{for}\;R_{j}<R_{\ell j},$$

where (*R*_ℓ*j*_,*Θ*_ℓ*j*_) is the polar form of the vector **x**^*j*^−**x**^ℓ^. Using the above and (2.9) we can impose the boundary conditions (2.8) to arrive at the following system of equations

2.11 $$A_{j}^{m}+I_{j}\;e^{im(\pi /2-\theta_{\mathrm{in}})}+\sum_{n=-\infty }^{\infty }\sum_{\overset{\ell =1}{\ell \neq j}}^{N}A_{\ell }^{n}Z_{\ell }^{n}\;e^{i(n-m)\Theta_{\ell j}}H_{n-m}(kR_{\ell j})=0,$$

for *j*=1,…,*N* and all integers *m*. Furthermore, the coefficients associated with the pressure inside the cylinder (2.7) are then given by

2.12 $$B_{j}^{m}=\frac{A_{j}^{m}}{J_{m}(k_{j}a_{j})}[Z_{j}^{m}H_{m}(ka_{j})-J_{m}(ka_{j})]$$

and subsequently, the field *u*(*x*,*y*) is entirely prescribed.

In any given material, it is impossible to know the exact position and properties of all constituent particles. Our goal is, therefore, to solve (2.11) not for one particular configuration of scatterers, but instead to calculate the average value of the coefficients $A_{j}^{m}$, denoted by $\left\langle A_{j}^{m}\right\rangle$, from which we can calculate an effective wavenumber and reflection. Note that the *effective* field describes the *ensemble-averaged* field that is usually measured in an acoustic experiment, as the receiver face is typically much larger than the particles and the distance between them [1,39]. In our case, we obtain an ensemble average by averaging over all particle configurations and all the material properties of the particles. This approach is general and can be tailored to different scenarios, e.g. when detailed information *is* known about the particle material properties.

## 3. Averaged multiple scattering

For an introduction to ensemble averaging of multiple scattering, see [2,40], where the result for a classical dilute isotropic mixture was determined. Here we present a brief self-contained explanation tailored to multi-species.

Consider a configuration of *N* circular cylinders centred at **x**_1_,**x**_2_,…,**x**_*N*_ with the scattering properties **s**_1_,**s**_2_,…,**s**_*N*_, where **s**_*j*_ denotes the properties of the *j*th cylinder, i.e. here these are **s**_*j*_=(*a*_*j*_,*ρ*_*j*_,*c*_*j*_). Each **x**_*j*_ is in the region $R_{N}$, where $n=N/|R_{N}|$ is the total number density and $|R_{N}|$ is the area of $R_{N}$. The properties **s**_*j*_ are taken from the set S. For example, we could have S=[0,1]×[1,2]×[100,200], so that *a*_*j*_∈[0,1], *ρ*_*j*_∈[1,2] and *c*_*j*_∈[100,200].

The probability of the cylinders being in a specific configuration is given by the probability density function *p*({**x**_1_,**s**_1_},{**x**_2_,**s**_2_},…,{**x**_*N*_,**s**_*N*_}). Using the compact notation ***Λ***_*i*_={**x**_*i*_,**s**_*i*_} to denote the properties of the *i*-th cylinder, it follows that

3.1 $$\int p(\Lambda_{1})\;d\Lambda_{1}=\iint p(\Lambda_{1},\Lambda_{2})\;d\Lambda_{1}\;d\Lambda_{2}=\cdots =1,$$

where each integral is taken over *both* $R_{N}$ (for **x**_*j*_) and S (for **s**_*j*_) with *d****Λ***_*j*_=*d***x**_*i*_ *d***s**_*i*_. Note that *p*(***Λ***_1_,***Λ***_2_) is the probability of one cylinder having the properties ***Λ***_1_ and another having the properties ***Λ***_2_, when the properties of all the remaining *N*−2 cylinders are unknown. And as the cylinders are indistinguishable: *p*(***Λ***_1_,***Λ***_2_)=*p*(***Λ***_2_,***Λ***_1_). Furthermore, we have

3.2a $$p(\Lambda_{1},\ldots ,\Lambda_{N})=p(\Lambda_{j})p(\Lambda_{1},\ldots ,\Lambda_{N}\;|\;\Lambda_{j})$$

and

3.2b $$p(\Lambda_{1},\ldots ,\Lambda_{N}\;|\;\Lambda_{j})=p(\Lambda_{\ell }\;|\;\Lambda_{j})p(\Lambda_{1},\ldots ,\Lambda_{N}\;|\;\Lambda_{\ell },\Lambda_{j}),$$

where *p*(***Λ***_1_,…,***Λ***_*N*_ | ***Λ***_*j*_) is the conditional probability of having cylinders with the properties ***Λ***_1_,…,***Λ***_*N*_ (not including ***Λ***_*j*_), given that the *j*th cylinder has the properties ***Λ***_*j*_. Likewise, *p*(***Λ***_1_,…,***Λ***_*N*_ | ***Λ***_ℓ_,***Λ***_*j*_) is the conditional probability of having cylinders with the properties ***Λ***_1_,…,***Λ***_*N*_ (not including ***Λ***_ℓ_ and ***Λ***_*j*_) given that there are already two cylinders present, with properties ***Λ***_ℓ_ and ***Λ***_*j*_.

Given some function *F*(***Λ***_1_,…,***Λ***_*N*_), we denote its average, or *expected value*, by

3.3 $$\langle F\rangle =\int \cdots \int F(\Lambda_{1},\ldots ,\Lambda_{N})p(\Lambda_{1},\ldots ,\Lambda_{N})\;d\Lambda_{1}\ldots d\Lambda_{N}.$$

If we fix the location and properties of the *j*th cylinder, ***Λ***_*j*_ and average over all the properties of the other cylinders, we obtain a *conditional average* of *F* given by

3.4 $${\left\langle F\right\rangle }_{\Lambda_{j}}=\int \ldots \int F(\Lambda_{1},\ldots ,\Lambda_{N})p(\Lambda_{1},\ldots ,\Lambda_{N}\;|\;\Lambda_{j})\;d\Lambda_{1}\ldots \Lambda_{N},$$

where we do not integrate over ***Λ***_*j*_. The average and conditional averages are related by

3.5 $$\langle F\rangle =\int {\left\langle F\right\rangle }_{\Lambda_{j}}p(\Lambda_{j})\;d\Lambda_{j}\;\text{and}\;{\left\langle F\right\rangle }_{\Lambda_{j}}=\int {\left\langle F\right\rangle }_{\Lambda_{j}\Lambda_{\ell }}p(\Lambda_{\ell })\;d\Lambda_{\ell },$$

where 〈*F*〉_***Λ***_ℓ_***Λ***_*j*__ is the conditional average when fixing both ***Λ***_*j*_ and ***Λ***_ℓ_, and 〈*F*〉_***Λ***_ℓ_***Λ***_*j*__=〈*F*〉_***Λ***_*j*_***Λ***_ℓ__.

Returning to the task of obtaining effective properties for a multi-species medium, we multiply the system (2.11) by *p*(***Λ***_2_,…,***Λ***_*N*_|***Λ***_1_) and average over ***Λ***_2_,…,***Λ***_*N*_, to reach

$$\begin{array}{rl} & \;\sum_{n=-\infty }^{\infty }\sum_{\ell =2}^{N}\int {\left\langle A_{\ell }^{n}\right\rangle }_{\Lambda_{\ell }\Lambda_{1}}Z^{n}({\text{s}}_{\ell })\;e^{i(n-m)\Theta_{\ell 1}}H_{n-m}(kR_{\ell 1})p(\Lambda_{\ell }|\Lambda_{1})\;d\Lambda_{\ell }\\ & \;\;+{\left\langle A_{1}^{m}\right\rangle }_{\Lambda_{1}}+I_{1}\;e^{im(\pi /2-\theta_{\mathrm{in}})}=0, \end{array}$$

where, without loss of generality, we have chosen *j*=1, used the conditional average definition (3.2b) and defined $Z^{n}({\text{s}}_{\ell }):=Z_{\ell }^{n}$ to make the dependency on **s**_ℓ_ explicit. To further simplify the above, note that all terms in the sum over ℓ give the same value. That is, the terms in the integrand depend on ℓ solely through the dummy variable ***Λ***_ℓ_. In particular, the probability distribution is the same for each cylinder, and if ***Λ***_2_=***Λ***_*l*_, then ${\left\langle A_{\ell }^{n}\right\rangle }_{\Lambda_{\ell }\Lambda_{1}}={\left\langle A_{2}^{n}\right\rangle }_{\Lambda_{2}\Lambda_{1}}$, because equation (2.11) uniquely determines the coefficients $A_{\ell }^{n}$ from the position and scattering properties ***Λ***_ℓ_. We use this to obtain

3.6 $$\begin{array}{rl} & \;\sum_{n=-\infty }^{\infty }(N-1)\int {\left\langle A_{2}^{n}\right\rangle }_{\Lambda_{2}\Lambda_{1}}Z^{n}({\text{s}}_{2})\;e^{i(n-m)\Theta_{21}}H_{n-m}(kR_{21})p(\Lambda_{2}\;|\;\Lambda_{1})\;d\Lambda_{2}\\ & \;\;+{\left\langle A_{1}^{m}\right\rangle }_{\Lambda_{1}}+I_{1}\;e^{im(\pi /2-\theta_{\mathrm{in}})}=0. \end{array}$$

Our aim is to solve the system above for ${\left\langle A_{1}^{m}\right\rangle }_{\Lambda_{1}}$; however, this requires that we make assumptions about *p*(***Λ***_2_ | ***Λ***_1_) and ${\left\langle A_{2}^{n}\right\rangle }_{\Lambda_{2},\Lambda_{1}}$. These approximations are discussed in §3a; however, for the moment, we assume that an appropriate substitution has been imposed.

With ${\left\langle A_{1}^{m}\right\rangle }_{\Lambda_{1}}$, we can calculate the average total pressure (incident plus scattered), measured at some position **x** outside $R_{N}$, by averaging (2.6) to obtain

3.7 $$\langle u(x,y)\rangle =u_{\mathrm{in}}(x,y)+\sum_{j=1}^{N}\int \ldots \int u_{j}(R_{j},\Theta_{j})p(\Lambda_{1},\ldots ,\Lambda_{N})\;d\Lambda_{1}\ldots d\Lambda_{N},$$

where 〈*u*_in_(*x*,*y*)〉=*u*_in_(*x*,*y*) because the incident field is independent of the scattering configuration. We can then rewrite the average outgoing wave *u*_*j*_ by fixing the properties of the *j*th cylinder ***Λ***_*j*_ and using equation (3.2a) to reach

3.8 $$\langle u(x,y)\rangle -u_{\mathrm{in}}(x,y)=\sum_{j=1}^{N}\int {\left\langle u_{j}(R_{j},\Theta_{j})\right\rangle }_{\Lambda_{j}}p(\Lambda_{j})\;d\Lambda_{j}=N\int {\left\langle u_{1}(R_{1},\Theta_{1})\right\rangle }_{\Lambda_{1}}p(\Lambda_{1})\;d\Lambda_{1}.$$

Likewise, for the conditionally averaged scattered field (2.4) measured at **x**, we obtain

3.9 $${\left\langle u_{1}(R_{1},\Theta_{1})\right\rangle }_{\Lambda_{1}}=\sum_{m=-\infty }^{\infty }{\left\langle A_{1}^{m}\right\rangle }_{\Lambda_{1}}Z^{m}({\text{s}}_{1})H_{m}^{\left(1\right)}(kR_{1})\;e^{im\Theta_{1}}.$$

We use the above to calculate the reflection from a halfspace in §7a and to obtain (1.2). To proceed we need to solve the system (3.6) and, in line with existing approaches, we do this by making statistical approximations.

### (a) Statistical approximations

In order to solve (3.6) for ${\left\langle A_{1}^{n}\right\rangle }_{\Lambda_{1}}$, we need an approximation for ${\left\langle A_{2}^{n}\right\rangle }_{\Lambda_{2},\Lambda_{1}}$ and the pair distribution *p*(***Λ***_2_ | ***Λ***_1_). In this work, we adopt the standard closure approximation for single species, but extended to multi-species, the QCA [5,6]:

3.10 $${\left\langle A_{2}^{n}\right\rangle }_{\Lambda_{2}\Lambda_{1}}\approx {\left\langle A_{2}^{n}\right\rangle }_{\Lambda_{2}}.$$

This approximation still makes sense for multi-species because it replaces the dependence of ${\left\langle A_{2}^{n}\right\rangle }_{\Lambda_{2},\Lambda_{1}}$ in ***Λ***_1_ by its expected value in ***Λ***_1_. Note also that the expected difference in ***Λ***_2_:

$$\int [{\left\langle A_{2}^{n}\right\rangle }_{\Lambda_{2}\Lambda_{1}}-{\left\langle A_{2}^{n}\right\rangle }_{\Lambda_{2}}]p(\Lambda_{2})\;d\Lambda_{2}={\left\langle A_{2}^{n}\right\rangle }_{\Lambda_{1}}-\langle A_{2}^{n}\rangle \approx 0,$$

for a large number of scatterers.

Using QCA, we introduce the notation

3.11 $$A^{n}({\text{x}}_{j},{\text{s}}_{j})={\left\langle A_{j}^{n}\right\rangle }_{\Lambda_{j}}\;\text{and}\;A^{n}({\text{x}}_{j},{\text{s}}_{j})={\left\langle A_{j}^{n}\right\rangle }_{\Lambda_{j}\Lambda_{k}},\;\text{for}\;k\neq j.$$

Next, we determine a suitable approximation for the pair distribution *p*(***Λ***_2_|***Λ***_1_), beginning with (3.2a) to write

3.12 $$p(\Lambda_{2}\;|\;\Lambda_{1})={\left[p(\Lambda_{1})\right]}^{-1}p(\Lambda_{1},\Lambda_{2}).$$

For clarity, we introduce the spatial random variables **X**_1_,**X**_1_,…,**X**_*N*_ and the scattering property random variables **S**_1_,**S**_1_,…,**S**_*N*_, and write probability density functions in the form, e.g.

3.13 $$p(\Lambda_{1},\ldots ,\Lambda_{N})=P({\text{X}}_{1}={\text{x}}_{1},\ldots ,{\text{X}}_{N}={\text{x}}_{N},{\text{S}}_{1}={\text{s}}_{1},\ldots ,{\text{S}}_{N}={\text{s}}_{N}).$$

In the first instance, we assume the random uniform distribution

3.14 $$p(\Lambda_{1})=\frac{1}{|R_{N}|}P({\text{S}}_{1}={\text{s}}_{1}),$$

where *P*(**S**_1_=**s**_1_) is the probability density in S that the particle will have scattering property **s**_1_. The above assumes that $P({\text{X}}_{1}={\text{x}}_{1}\;|\;{\text{S}}_{1}={\text{s}}_{1})=|R_{N}{|}^{-1}$, i.e. that the position **x**_1_ of the cylinder is independent of the scattering property **s**_1_. This is not always the case, for example, depending on the size of the cylinder, some positions near the boundary of $R_{N}$ may be infeasible. However, these boundary effects are negligible when taking the limit $|R_{N}|\to \infty$.

For the remaining distribution in (3.12), we use

3.15 $$p(\Lambda_{1},\Lambda_{2})=P({\text{S}}_{1}={\text{s}}_{1},{\text{S}}_{2}={\text{s}}_{2})P({\text{X}}_{1}={\text{x}}_{1},{\text{X}}_{2}={\text{x}}_{2}\;|\;{\text{S}}_{1}={\text{s}}_{1},{\text{S}}_{2}={\text{s}}_{2}),$$

followed by

3.16 $$P({\text{S}}_{1}={\text{s}}_{1},{\text{S}}_{2}={\text{s}}_{2})=P({\text{S}}_{1}={\text{s}}_{1})P({\text{S}}_{2}={\text{s}}_{2}),$$

which assumes that the scattering properties are statistically independent. Next, we assume that the cylinders are equally likely to be anywhere but do not overlap (a hole correction correlation [20]), to write

3.17 $$P({\text{X}}_{1}={\text{x}}_{1},{\text{X}}_{2}={\text{x}}_{2}\;|\;{\text{S}}_{1}={\text{s}}_{1},{\text{S}}_{2}={\text{s}}_{2})=\left\{ \begin{array}{ll} 0 & \text{if}\;R_{21}\leq a_{21},\\ |R_{N}{|}^{-2} & \text{if}\;R_{21}>a_{21}, \end{array}\right.$$

where *R*_21_:=∥**x**_1_−**x**_2_∥, *a*_21_=*b*_1_+*b*_2_ for some *b*_1_≥*a*_1_ and some *b*_2_≥*a*_2_, and *b*_1_ is the radius of exclusion around **x**_1_ which is usually chosen to be proportional to the radius *a*_1_. Note that when integrating (3.17) above in **x**_1_ and **x**_2_, we obtain $|R_{N}{|}^{-2}(|R_{N}{|}^{2}-\pi a_{21}^{2})\approx 1$ for $R_{N}\gg a_{21}^{2}$.

Ultimately, substituting (3.16) and (3.17) into (3.15) in tandem with (3.14) leads to the pair distribution

3.18 $$p(\Lambda_{2}\;|\;\Lambda_{1})=\frac{1}{|R_{N}|}p({\text{s}}_{2})H(R_{21}-a_{21}),$$

where *H*(*x*) denotes the Heaviside function, under the assumption $|R_{N}|\gg a_{21}^{2}$. In the next section, we will the approximations (3.11) and (3.18) to solve the system in (3.6) for ${\left\langle A_{1}^{n}\right\rangle }_{\Lambda_{1}}$.

We now include a discussion of other commonly used pair distributions. We remark that for densely packed scatterers, other pair distributions [41] are preferred and take the form

3.19 $$P({\text{X}}_{1}={\text{x}}_{1},{\text{X}}_{2}={\text{x}}_{2}\;|\;{\text{S}}_{1}={\text{s}}_{1},{\text{S}}_{2}={\text{s}}_{2})=\left\{ \begin{array}{ll} 0 & \text{if}\;R_{21}\leq a_{21},\\ \frac{1+\chi (R_{21}\;|\;{\text{s}}_{1},{\text{s}}_{2})}{|R_{N}{|}^{2}} & \text{if}\;R_{21}>a_{21}, \end{array}\right.$$

where

3.20 $$\int_{R_{N}}\int_{R_{N}}\chi (R_{21}\;|\;{\text{s}}_{1},{\text{s}}_{2})\;d{\text{x}}_{1}\;d{\text{x}}_{2}=0.$$

To calculate the effective wavenumber for the pair-correlation (3.19), a common choice is to assume that the scatterers are uniformly randomly distributed, which leads to

3.21 $$\chi (R_{21}\;|\;{\text{s}}_{1},{\text{s}}_{2})\approx 0\;\text{for}\;R_{21}>{\bar{a}}_{21}>2a_{21},$$

used by Linton & Martin [5], Section D, [42], [43], eqn (27), where ${\bar{a}}_{21}$ is some distance large enough for the scatterers at **x**_1_ and **x**_2_ to no longer effect each other. One popular choice for *χ* is the Percus–Yevick function, which assumes all scatterers are uniformly randomly distributed [44], though *χ* can also be used to specify if some species are more likely to be closer or further apart.

In this work, we set *χ*=0 for simplicity (unless otherwise stated), but also because it is not clear that the error introduced by using *χ*=0 is in any way greater than the error committed due to QCA (3.10). Both the hole correction (3.17) and QCA (3.10) make similar assumptions: for *R*_21_>*a*_21_, the hole correction replaces *p*(***Λ***_2_ | ***Λ***_1_) with its expected value in ***Λ***_1_:

$$p(\Lambda_{2}\;|\;\Lambda_{1})\approx \int p(\Lambda_{2}\;|\;\Lambda_{1})p(\Lambda_{1})\;d\Lambda_{1}=p(\Lambda_{2}),$$

just as QCA (3.10) assumes that ${\left\langle A_{2}^{n}\right\rangle }_{\Lambda_{2}\Lambda_{1}}\approx {\left\langle A_{2}^{n}\right\rangle }_{\Lambda_{2}}$. Similarly for *R*_21_≤*a*_21_ we would set both *p*(***Λ***_2_ | ***Λ***_1_)=0 and ${\left\langle A_{2}^{n}\right\rangle }_{\Lambda_{2}\Lambda_{1}}=0$ for QCA and hole correction. Another reason to set *χ*=0 is because we are interested in the limit for small n. In this limit, it is expected that *χ*→0 when n→0 for uniformly distributed scatterers, which in turn indicates that the contribution of *χ* to the effective wave is smaller than $n^{2}$ [5,45].

### (b) Infinitely many cylinders in the halfspace

In preceding sections, we considered a finite number of scatterers in a bounded domain $R_{N}$. Now we consider the limit N→∞ and where the region $R_{N}$ tends to the halfspace *x*>0. We will follow [5] and limit the cylinders to the halfspace *x*>0, as it allows us to avoid divergent integrals, such as those in [46], e.g. between their eqn (32) and (33).

Substituting the approximations (3.18) and (3.11) into the governing system (3.6) leads to

3.22 $$\begin{array}{rl} & \frac{N-1}{|R_{N}|}\sum_{n=-\infty }^{\infty }\int_{S}\int_{\overset{R_{N}}{R_{21}>a_{21}}}A^{n}({\text{x}}_{2},{\text{s}}_{2})\;e^{i(n-m)\Theta_{21}}H_{n-m}(kR_{21})\;d{\text{x}}_{2}\;d{\text{s}}_{2}^{n}\\ & \;\;+A^{m}({\text{x}}_{1},{\text{s}}_{1})+I_{1}\;e^{im(\pi /2-\theta_{\mathrm{in}})}=0,\;\text{for}\;x_{1}>0, \end{array}$$

where for brevity, we write

3.23 $$d{\text{s}}_{2}^{n}=Z^{n}({\text{s}}_{2})p({\text{s}}_{2})\;d{\text{s}}_{2},$$

with *p*(**s**_2_)=*P*(**S**_2_=**s**_2_). By taking the limits N→∞ and $\lim_{N\to \infty }R_{N}=\{(x_{1},x_{2}):x_{2}>0\}$, while fixing the number density $n=N/|R_{N}|$, equation (3.22) takes the form

3.24 $$\begin{array}{rl} & n\sum_{n=-\infty }^{\infty }\int_{S}\int_{\overset{x_{2}>0}{R_{21}>a_{21}}}A^{n}({\text{x}}_{2},{\text{s}}_{2})\;e^{i(n-m)\Theta_{21}}H_{n-m}(kR_{21})\;d{\text{x}}_{2}\;d{\text{s}}_{2}^{n}\\ & \;\;+A^{m}({\text{x}}_{1},{\text{s}}_{1})+I_{1}\;e^{im(\pi /2-\theta_{\mathrm{in}})}=0,\;\text{for}\;x_{1}>0, \end{array}$$

which represents the governing system for our semi-infinite multiple-species problem.

Incidentally, when all cylinders are identical this system reduces to eqn (54) in [5], that is when *p*(**s**_2_)=*δ*(*a*_2_−*a*)*δ*(*c*_2_−*c*)*δ*(*ρ*_2_−*ρ*) in (3.23), where *δ*(*x*) represents Dirac’s delta function.

## 4. Effective wavenumber

To solve the system (3.24), first we use the symmetry of the problem to rewrite

4.1 $$A^{m}(x,y,\text{s})=A^{m}(x,0,\text{s})\;e^{i\beta y},$$

that is, if $A^{m}$ is a solution to (3.24), then so is $A_{0}^{m}$ defined by $A_{0}^{m}(x,y,\text{s})=A^{m}(x,y-y^{\prime },\text{s})\;e^{i\beta y^{\prime }}$ for every *y*′, then taking *y*′=*y* we see that (4.1) is also a solution, recalling that *I*_1_=*e*^i*αx*+*iβy*^ and

4.2 $$\alpha =k\cos\theta_{\mathrm{in}}\;\text{and}\;\beta =k\sin\theta_{\mathrm{in}}.$$

Sufficiently far away from the boundary, say $x>\bar{x}$, we assume a plane wave ansatz

4.3 $$A^{m}(x,y,\text{s})=i^{m}\;e^{-im\theta_{*}}A_{*}^{m}(\text{s})\;e^{i{\text{k}}_{*}\cdot \text{x}},\;\text{for}\;x>\bar{x},$$

where the factor *i*^*m*^ *e*^−imθ_*_^ is introduced for later convenience. We could have for generality considered a sum of plane waves, but for low number density this is unnecessary, as we would find a unique k_*_ for a halfspace.

Equating (4.1) and (4.3), for $x>\bar{x}$, we obtain Snell’s Law

4.4 $$k_{*}\sin\theta_{*}=k\sin\theta_{\mathrm{in}}\;\text{with}\;{\text{k}}_{*}=(\alpha_{*},\beta ):=k_{*}(\cos\theta_{*},\sin\theta_{*}),$$

noting that both θ_*_ and k_*_ are complex numbers. We also require that Im α_*_>0, so that the integral over **x**_2_ in (3.24) converges.

In appendix B, we present the derivation for the system below, which is obtained by substituting (4.1) and (4.3) into (3.24). In the process we establish that k_*_≠*k*, and find that there is no restriction on the length $\bar{x}$, a fact we use to calculate the reflected wave. The result is that (3.24) reduces to the system

4.5 $$A_{*}^{m}({\text{s}}_{1})+2n\pi \sum_{n=-\infty }^{\infty }\int_{S}A_{*}^{n}({\text{s}}_{2})\left[\frac{N_{n-m}(ka_{12},k_{*}a_{12})}{k^{2}-k_{*}^{2}}+X_{*}\right]d{\text{s}}_{2}^{n}=0$$

and

4.6 $$\sum_{n=-\infty }^{\infty }e^{in(\theta_{\mathrm{in}}-\theta_{*})}\int_{S}A_{*}^{n}({\text{s}}_{2})\;d{\text{s}}_{2}^{n}=e^{i(\alpha -\alpha_{*})\bar{x}}(\alpha_{*}-\alpha )\left[\frac{\alpha i}{2n}+b(\bar{x})\right],$$

in terms of the unknown parameters $A_{*}^{n}({\text{s}}_{2})$ and k_*_, where

4.7 $$\begin{array}{rl} b(\bar{x}) & \;={\left(-i\right)}^{n-1}\sum_{n=-\infty }^{\infty }e^{in\theta_{\mathrm{in}}}\int_{S}\int_{0}^{\bar{x}}A^{n}(x_{2},0,{\text{s}}_{2})\;e^{-i\alpha x_{2}}\;dx_{2}\;d{\text{s}}_{2}^{n}, \end{array}$$

4.8 $$\begin{array}{rl} N_{n}(x,y) & \;=xH_{n}^{\prime }(x)J_{n}(y)-yH_{n}(x)J_{n}^{\prime }(y) \end{array}$$

and $X_{*}=0$, as we have assumed *hole correction* (3.17). For a more general pair distribution (3.19), we obtain

4.9 $$X_{*}=\int_{a_{21}<R<{\bar{a}}_{21}}H_{n-m}(kR)J_{n-m}(k_{*}R)\chi (R\;|\;{\text{s}}_{1},{\text{s}}_{2})R\;dR,$$

where further details may be found in appendix Ba. We also remark that equation (4.5) reduces to ([5], eqn (87),[46], eqn (33)) for a single particle species.

To determine the effective wavenumber k_*_ we need only use (4.5). That is, the solution k_*_ is the one that leads to non-trivial solutions for the function $A_{*}^{m}$. On the other hand, if $A_{*}^{m}({\text{s}}_{1})$ is a solution to (4.5), then so is $cA_{*}^{m}({\text{s}}_{1})$ for any constant *c*. To completely determine $A_{*}^{m}({\text{s}}_{1}),$ we need to use (4.5) and (4.6).

Next, we determine closed-form estimates for k_*_ from (4.5) and determine the corresponding coefficients $A_{*}^{m}({\text{s}}_{1})$ for low number density in §7.

### (a) Explicit expressions for k_*_ via expansions in the number density

We now consider the expansions

4.10 $$k_{*}^{2}∼K_{*0}+K_{*1}n+K_{*2}n^{2}\;\text{and}\;A_{*}^{m}∼A_{*0}^{m}+A_{*1}^{m}n+A_{*2}^{m}n^{2},$$

where we use ∼ to denote an asymptotic expansion in n, which is formally equivalent to an expansion in volume fraction. We show in appendix Bb how substituting the above into equation (4.5) leads to

4.11 $$k_{*}^{2}=k^{2}-4ni\langle \;f_{\circ }\rangle (0)-4n^{2}i\langle \;f_{\circ \circ }\rangle (0)+O(n^{3}),$$

where we assumed K_*0_=*k*^2^ , though we deduce this in §7. Here we have

4.12 $$f_{\circ }(\theta ,{\text{s}}_{1})=-\sum_{n=-\infty }^{\infty }e^{in\theta }Z^{n}({\text{s}}_{1})\;\text{and}\;\langle \;f_{\circ }\rangle (\theta )=-\sum_{n=-\infty }^{\infty }e^{in\theta }\int_{S}d{\text{s}}_{1}^{n}$$

and we introduce the multiple scattering pattern

4.13 $$\langle \;f_{\circ \circ }\rangle (\theta )=-\pi \sum_{n,m=-\infty }^{\infty }\int_{S}e^{in\theta }a_{12}^{2}d_{n-m}(ka_{12})\;d{\text{s}}_{1}^{n}\;d{\text{s}}_{2}^{m},$$

where $d{\text{s}}_{1}^{n}=Z^{n}({\text{s}}_{1})p({\text{s}}_{1})\;d{\text{s}}_{1}$ and for convenience we define

4.14 $$d_{m}(x)=J_{m}^{\prime }(x)H_{m}^{\prime }(x)+\left(1-{\left(\frac{m}{x}\right)}^{2}\right)J_{m}(x)H_{m}(x).$$

We remark that *f*_°_ corresponds to the far-field scattering pattern for a single circular cylinder. This is evident by taking *N*=1 in (2.11), which leads to

4.15 $$A_{1}^{m}=-i^{m}\;e^{-im\theta_{\mathrm{in}}}\;e^{i{\text{x}}_{1}\cdot \text{k}}$$

and from (2.4) gives

$$\lim_{R_{1}\to \infty }u_{1}∼\sqrt{\frac{2}{\pi kR_{1}}}f_{\circ }(\Theta_{1}-\theta_{\mathrm{in}},{\text{s}}_{1})\;e^{ikR_{1}-i\pi /4}.$$

We can interpret the terms on the right-hand side of (4.11) in the following way: the first *k*^2^ corresponds to the incident wave, the second $4ni\langle \;f_{\circ }\rangle (0)$ is the contribution from the incident wave scattered once from every cylinder (so-called ‘single scattering’) and the last $4n^{2}i\langle \;f_{\circ \circ }\rangle (0)$ is the contribution of this scattered wave being re-scattered by every cylinder (so-called ‘multiple scattering’).

We can further specialize the wavenumber (4.11) by considering wavelengths 2*π*/*k* larger than the largest cylinder radius, or more precisely

4.16 $$ka_{*}:=\max_{{\text{s}}_{1}}\{ka_{11}p^{2}({\text{s}}_{1})\}\ll 1,$$

which leads to

4.17 $$k_{*}^{2}∼k^{2}-4ni\langle \;f_{\circ }\rangle (0)+\frac{8n^{2}}{\pi k^{2}}\int_{0}^{\pi }\cot\left(\frac{\theta }{2}\right)\frac{d}{d\theta }{\left[\langle \;f_{\circ }\rangle (\theta )\right]}^{2}\;d\theta +O(k^{4}a_{*}^{4}\log(ka_{*})),$$

where the integral converges because 〈 *f*_°_〉′(0)=0. This expression and the derivation is analogous to that given in ([5], eqn (86)) for a single species. Although the sums in (4.11) converge quickly for small ka_*_, the form (4.17) is convenient as it is written in terms of the far-field scattering pattern 〈 *f*_°_〉.

An alternative approach [47] that is very useful in the context of low-frequency propagation is to take the quasi-static limit of the system (4.5). For small *ka*, the monopole and dipole scattering coefficients are both *O*((*ka*)^2^), which are the only contributions to the effective bulk modulus and density, respectively. Following the approach in [47], it is straightforward to show that for the *N*-species case, where the *n*th species has volume fraction *ϕ*_*n*_, bulk modulus *K*_*n*_ and density *ρ*_*n*_, the effective bulk modulus K_*_ and density ρ_*_ take the form

4.18 $$K_{*}^{-1}=K^{-1}(1-\phi )+\sum_{n=1}^{N}K_{n}^{-1}\phi_{n}\;\mathrm{and}\;\rho_{*}=\rho \left(\frac{1+\sum_{n=1}^{N}D_{n}\phi_{n}}{1-\sum_{n=1}^{N}D_{n}\phi_{n}}\right),$$

where $\phi =\sum_{n}\phi_{n}$ and *D*_*n*_=(*ρ*_*n*_−*ρ*)/(*ρ*_*n*_+*ρ*).

Next, we explore how the expression (4.11) compares with other approaches by evaluating it numerically. In §7, we develop analytical expressions for the average scattering coefficient $A^{n}$ and expressions for the reflection coefficient from the inhomogeneous halfspace.

## 5. Two species of cylinders

**Figure 2.**
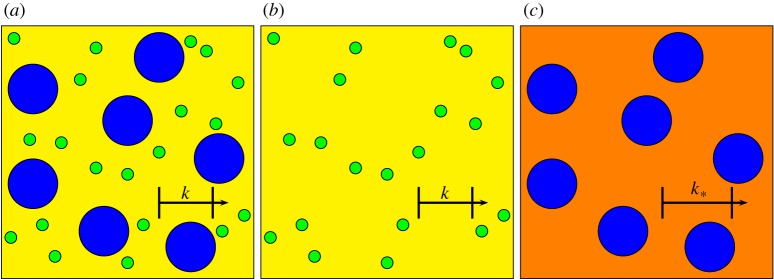
(*a*) Two-species material comprising large (blue) and small (green) inclusions in a background material (yellow) with incident wavenumber *k*, (*b*) one-species material comprising the small inclusions alone, and (*c*) one-species material with large cylinders alone in a background with incident wavenumber k_*_, which is the effective wavenumber of (*b*). (Online version in colour.)

In this section, we analytically compare two approaches to calculating the effective wavenumber of a multi-species material. The first self-consistent type method homogenizes the small cylinder distribution and then determines effective properties for a large cylinder distribution embedded in the homogenized background, as shown in figure 2. The second determines the multi-species result using the approach outlined in previous sections.

### (a) One small and one large species

We begin by assuming that there are only two species, *S* and *L*, that have constant wave speeds *c*_*S*_ and *c*_*L*_, densities *ρ*_*S*_ and *ρ*_*L*_, and number fractions $n_{S}$ and $n_{L}$, respectively. We assume that both types of cylinders have low volume fractions $\phi_{S}=\pi a_{S}^{2}n_{S}$ and $\phi_{L}=\pi a_{L}^{2}n_{L}$ and are proportional to one another *ϕ*_*S*_∝*ϕ*_*L*_, so we will discard $O(\phi^{3})$ terms, where *ϕ*=*ϕ*_*S*_+*ϕ*_*L*_ denotes the total volume filling fraction. Note that it is more precise to assume small *ϕ*, rather than a small number density, since *ϕ* is non-dimensional.

First, the effective wavenumber k_*S_ of a material at long wavelengths with only a single species of *S*-cylinders is obtained by simplifying (4.17), where the far-field pattern (4.12) is, therefore, just the *S* cylinder species, i.e. there is no integral over **s**_1_ and *p*(**s**_1_)=1. Assuming *c*_*S*_≠0 and *ρ*_*S*_≠0, we use ([8], eqn (24)) for small cylinder radius, which in our notation (recall $Z^{n}({\text{s}}_{\ell }):=Z_{\ell }^{n}$, where $Z_{\ell }^{n}$ is given in (2.5))

5.1 $$Z^{0}({\text{s}}_{S})=i\pi \frac{a_{S}^{2}k^{2}}{4}P+O(a_{S}^{4})\;\mathrm{and}\;Z^{1}({\text{s}}_{S})=Z^{-1}({\text{s}}_{S})=i\pi \frac{a_{S}^{2}k^{2}}{4}Q+O(a_{S}^{4}),$$

where

5.2 $$P=1-\frac{k_{S}^{2}\rho }{k^{2}\rho_{S}}\;\mathrm{and}\;Q=\frac{\rho -\rho_{S}}{\rho +\rho_{S}}.$$

Substituting the above into the simplified (4.17) leads to

5.3 $$\frac{k_{*S}}{k}=1-\frac{\phi_{S}}{2}(P+2Q)-\frac{\phi_{S}^{2}}{8}(2P^{2}-{\left(P+2Q\right)}^{2})+O(a_{S}^{2})+O(\phi^{3}),$$

after taking a Taylor series for small n for the square root. We also calculate the effective density ([8], eqn (1)) or refer to (4.18) with *N*=1, given by

5.4 $$\frac{\rho_{*S}}{\rho }=\frac{\rho +\rho_{S}-\phi_{S}(\rho -\rho_{S})}{\rho +\rho_{S}+\phi_{S}(\rho -\rho_{S})}=1-2\phi_{S}Q+O(a_{S}^{2})+O(\phi^{3}),$$

which is appropriate for the approximation (5.3), see [8] for more details.

Next, we determine the effective wavenumber for large scatterers embedded in a background described by k_*S_ and ρ_*S_. For this step, we introduce the notation *f*_°_(0,**s**_1_)=*f*_°_(0,**s**_1_,*ρ*,*k*), which expresses the problem in terms of density and wavenumber in place of density and wave speed. Consequently, from (5.4), we have

5.5 $$f_{\circ }(0,{\text{s}}_{L},\rho_{*S},k_{*S})=f_{\circ L}(0)-\phi_{S}\delta f_{LS}+O(a_{S}^{2})+O(\phi^{2})$$

with

5.6 $$\delta f_{LS}:=2\rho Q\partial_{\rho }f_{\circ }(0,{\text{s}}_{L},\rho ,k)+\frac{k}{2}(P+2Q)\partial_{k}f_{\circ }(0,{\text{s}}_{L},\rho ,k),$$

where we set *f*_°*L*_(0):=*f*_°_(0,**s**_*L*_,*ρ*,*k*).

To calculate the wavenumber k_*LS_ for the *L*-cylinders in a material with a wavenumber k_*S_, we use the formula (4.11) with *k* replaced by k_*S_, 〈 *f*_°_〉 replaced with *f*_°_(0,**s**_*L*_,ρ*_S_,k_*S_) above and keeping only the integrands, that is, removing the integrals over the multi-species **s**_1_ and **s**_2_, to arrive at

5.7 $$\begin{array}{rl} k_{*LS}^{2} & \;=k_{*S}^{2}+4i\frac{\phi_{L}^{2}}{\pi a_{L}^{4}}\sum_{n,p=-\infty }^{\infty }a_{LL}^{2}d_{p-n}(k_{*S}a_{LL})Z^{n}({\text{s}}_{L},\rho_{*S},k_{*S})Z^{p}({\text{s}}_{L},\rho_{*S},k_{TS})\\ & \;\;-\frac{4i\phi_{L}}{\pi a_{L}^{2}}f_{\circ }(0,{\text{s}}_{L},\rho_{*S},k_{*S})+O(\phi^{3}) \end{array}$$

5.8 $$\begin{array}{rl} & \;=k_{*S}^{2}+4i\frac{\phi_{L}^{2}}{\pi a_{L}^{4}}\sum_{n,p=-\infty }^{\infty }a_{LL}^{2}d_{p-n}(ka_{LL})Z^{n}({\text{s}}_{L},\rho ,k)Z^{p}({\text{s}}_{L},\rho ,k)\\ & \;\;-\frac{4i\phi_{L}}{\pi a_{L}^{2}}(f_{\circ L}(0)-\phi_{S}\delta f_{LS})+O(a_{S}^{2})+O(\phi^{3}), \end{array}$$

where we used $d{\text{s}}_{j}^{m}=Z^{m}({\text{s}}_{j})p({\text{s}}_{j})\;d{\text{s}}_{j}$. The above is an attempt to calculate the multi-species wavenumber by using only the single-species formula. However, the term of order *O*(*ϕ*_*L*_*ϕ*_*S*_) in the above does not agree with (4.11), even in the limit *a*_*S*_→0, as we show next.

For only two species of cylinders, and assuming the cylinders are uniformly distributed, the probability density function for the scattering properties becomes

5.9 $$p(\text{s})=\frac{n_{S}}{n}\delta (a-a_{S})\delta (c-c_{S})\delta (\rho -\rho_{S})+\frac{n_{L}}{n}\delta (a-a_{L})\delta (c-c_{L})\delta (\rho -\rho_{L}),$$

which when substituted into (4.11) leads to the multi-species result

5.10 $$\begin{array}{rl} k_{*}^{2} & \;=k^{2}-4i(n_{S}f_{\circ S}(0)+n_{L}f_{\circ L}(0))+4n_{S}^{2}a_{SS}^{2}\pi i\sum_{n,p=-\infty }^{\infty }d_{p-n}(ka_{SS})Z^{p}({\text{s}}_{S})Z^{n}({\text{s}}_{S})\\ & \;\;+8n_{S}n_{L}a_{SL}^{2}\pi i\sum_{n,p=-\infty }^{\infty }d_{p-n}(ka_{SL})Z^{p}({\text{s}}_{S})Z^{n}({\text{s}}_{L})\\ & \;\;+4n_{L}^{2}a_{LL}^{2}\pi i\sum_{n,p=-\infty }^{\infty }d_{p-n}(ka_{LL})Z^{p}({\text{s}}_{L})Z^{n}({\text{s}}_{L})+O(\phi^{3}), \end{array}$$

where we used *a*_*LS*_=*a*_*SL*_. Assuming that *a*_*S*_≪1 and using *a*_*LS*_=*b*_*S*_+*b*_*L*_, with *b*_*S*_≥*a*_*S*_ and *b*_*L*_≥*a*_*L*_, we expand *d*_*m*_ (4.14) as

5.11 $$d_{m}(ka_{SL})=d_{m}(kb_{L})+\frac{2b_{S}}{b_{L}}[J_{m}(kb_{L})H_{m}(kb_{L})-d_{m}(kb_{L})]+O(a_{S}^{2}),$$

where we use *b*_*S*_∝*a*_*S*_. Substituting the above, (5.1), (5.3) and (5.8), into (5.10) we obtain

5.12 $$k_{*}^{2}=k_{*LS}^{2}+\phi_{L}\phi_{S}\left[-\frac{4i}{\pi a_{L}^{2}}\delta f_{LS}+H_{0}+\frac{a_{S}}{a_{L}}H_{1}\right]+O(a_{S}^{2})+O(\phi^{3}),$$

where

5.13 $$G_{0}=\frac{8i}{\pi }\frac{b_{L}^{2}}{a_{L}^{2}}\sum_{n=-\infty }^{\infty }\sum_{p=-1}^{1}d_{p-n}(kb_{L})\frac{Z^{p}({\text{s}}_{S})}{a_{S}^{2}}Z^{n}({\text{s}}_{L})$$

and

5.14 $$G_{1}=\frac{16i}{\pi }\frac{b_{L}b_{S}}{a_{L}a_{S}}\sum_{n=-\infty }^{\infty }\sum_{p=-1}^{1}J_{p-n}(kb_{L})H_{p-n}(kb_{L})\frac{Z^{p}({\text{s}}_{S})}{a_{S}^{2}}Z^{n}({\text{s}}_{L}).$$

Note that *Z*^*p*^(**s**_*S*_)/*a*^2^_*S*_ converges when *a*_*S*_→0, see (5.1).

The terms in the brackets in (5.12) account for the interaction between the two types of cylinders, which is where the wavenumbers k_*LS_ (5.8) and k_*_ (5.12) differ. The leading-order error is non-vanishing even as the radius of the small species vanishes and is given by

$$\lim_{a_{S}\to 0}\{k_{*}-k_{*LS}\}\approx \left(G_{0}-\frac{4i}{\pi a_{L}^{2}}\delta f_{LS}\right)\phi_{S}\phi_{L}.$$

A numerical investigation of this limit is conducted in §6. The physical meaning of these two terms are quite different: *δf*_*LS*_ is the change in the far-field scattering pattern of the *L*-cylinders due to changing the background wavenumber from *k* to k_*S_, while *G*_0_ accounts for the multiple scattering between the *L* and *S*-cylinders, which becomes significant when both *ϕ*_*S*_ are *ϕ*_*L*_ are large. Ultimately, this means that k_*_≈k_*LS_ if either the *S* or *L*-cylinders are very dilute.

For comparison, we also give the two-dimensional version of eqn (23) of [28] given by

5.15 $$\begin{array}{rl} k_{*C}^{2} & \;=k^{2}-4in_{S}f_{S}(0)-4in_{L}f_{L}(0)+\frac{8n_{S}^{2}}{\pi k^{2}}\int_{0}^{\pi }\cot(\theta /2)\frac{d}{d\theta }{\left[f_{S}(\theta )\right]}^{2}\;d\theta \\ & \;\;+\frac{8n_{L}^{2}}{\pi k^{2}}\int_{0}^{\pi }\cot(\theta /2)\frac{d}{d\theta }{\left[f_{L}(\theta )\right]}^{2}\;d\theta \end{array}$$

and another commonly used approximation [48]

5.16 $$k_{*0}^{2}=k^{2}-4in_{S}f_{S}(0)-4in_{L}f_{L}(0),$$

describing the effective wavenumber for two species. However, the expression (5.15) is missing the interaction between the $n_{S}$ and $n_{L}$ species (the term $O(n_{S}n_{L})$) and is only valid for low frequency. Additionally, the estimate (5.16) ignores terms of the order $O(\phi^{2})$.

## 6. Numerical examples

In this section, we consider a selection of numerical examples to demonstrate the efficacy of (5.10) and other expressions. For k_*LS_, we use the exact formula (4.11) for one species and then equation (5.7). This way, k_*LS_ is valid for *S*-cylinders with approximately zero density such as air. In the graphs that follow we use

6.1 sound speed=ωRe k∗andattenuation=Im k∗,

where k_*_ will be replaced with k_*LS_ and k_*0_ depending on the context.

For reference, we provide Julia [49] code to calculate the effective wavenumbers.

### (a) Two-dimensional emulsion

**Figure 3.**
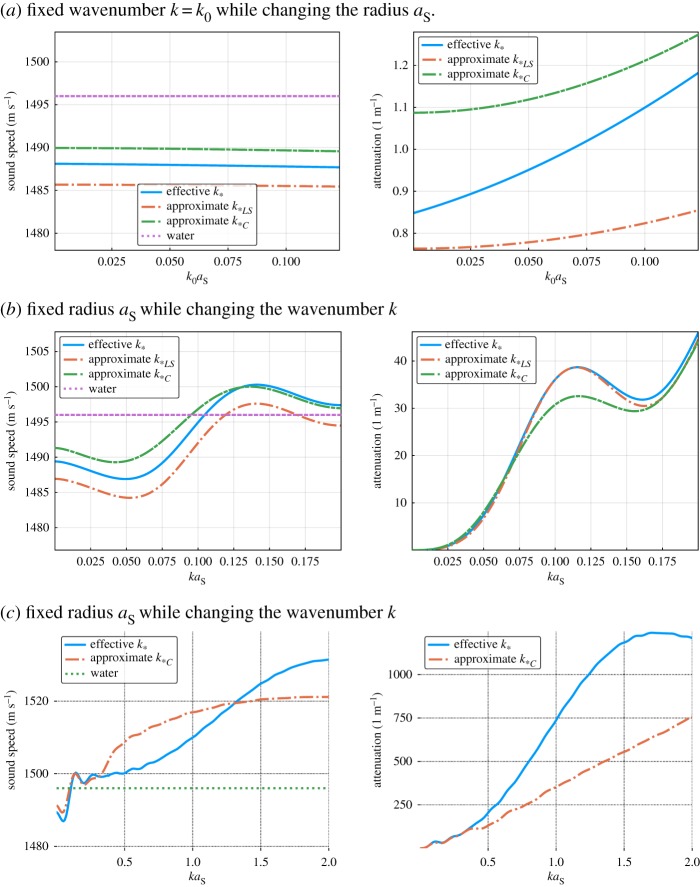
Comparison of sound speeds and attenuation using wavenumbers k_*C_ (5.15), k_*LS_ (5.7), and k_*_ (5.10) for the water and oil emulsion from table 2. Code to generate figure: https://github.com/arturgower/EffectiveWaves.jl/tree/master/examples/emulsion. (*a*) Fixed wavenumber *k*=*k*_0_ while changing the radius *a*_*S*_, (*b*) fixed radius *a*_*S*_ while changing the wavenumber *k* and (*c*) fixed radius *a*_*S*_ while changing the wavenumber *k*. (Online version in colour.)

Here we consider an emulsion composed of hexadecane (oil) and glycerol in water [50], table 2, where the glycerol forms very small inclusions. The graphs of figure 3*a* show how k_*_, k_*LS_ and k_*C_ differ when varying only *a*_*S*_ the radius of the glycerol inclusions, for a fixed angular frequency *ω*=*c*/*k*_0_≈3×10^6^ Hz. We observe that the difference between k_*_ and k_*LS_ persists even as *a*_*S*_→0, as expected according to (5.12). Meaning that, no matter how small the *S*-cylinders become, the larger cylinders *L* do not perceive the *S*-cylinders as a homogeneous material, in the naive way described in §5a.

**Table 2.** Material properties used to approximate an emulsion.

	density (kg m^−3^)	speed (*m* *s*^−1^)	radius (μm)	volume %
distilled water	*ρ*=998	*c*=1496	—	84%
hexadecane	*ρ*_*L*_=773	*c*_*L*_=1338	*a*_*L*_=250	*ϕ*_*L*_=11%
glycerol	*ρ*_*S*_=1260	*c*_*S*_=1904	*a*_*S*_=25	*ϕ*_*S*_=11%

In fig. 21 of [28], they observed that experimentally measured wave speeds were shifted in comparison to the k_*C_ predictions, even for low-frequency. We can see this same discrepancy in figure 3*b*, where the angular frequency is varied between 1 KHz<*ω*<12 MHz while the radius *a*_*S*_=25 μ*m* is fixed. This discrepancy is due to the terms of order $O(n_{L}n_{S})$ which are missing from k_*C_ (5.15) and k_*LS_ (5.10). Although all three wavenumbers are similar in figure 3*b*. The same is not true when we increase the frequency.

In figure 3*c*, we show how k_*C_, valid only for low-frequency, strays from the more accurate k_*_ as the frequency increases,^1^ where we did not include k_*LS_ as it is only valid for low frequency. There we can see that k_*C_ performs well up to about *ka*_*S*_=0.3, at which point *ka*_*L*_=3.0.

**Figure 4.**
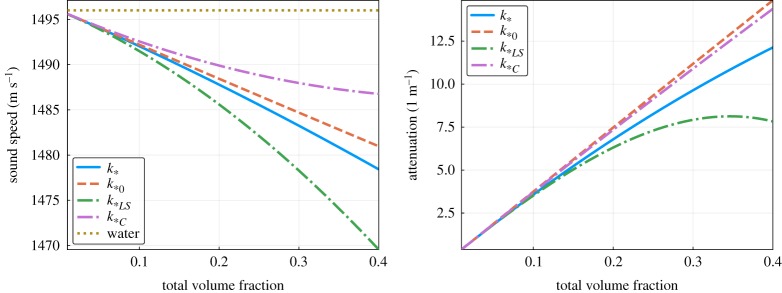
Comparison of sound speeds and attenuation calculated from the effective wavenumbers k_*C_ (5.15), k_*0_ (5.16), k_*LS_ (5.7) and the more accurate k_*_ (5.10), as the total volume fraction of the inclusions increases (for the emulsion shown in table 2 with *ka*_*S*_=0.5). Code: https://github.com/arturgower/EffectiveWaves.jl/tree/master/examples/emulsion. (Online version in colour.)

All the approximations k_*0_ (5.16), k_*LS_ (5.10) and k_*C_ (5.15) are missing second-order terms in the number density. In figure 4, we see the effect of these missing terms by varying the volume fraction while fixing *ω*=3×10^6^, or equivalently *ka*_*S*_=0.5. In the limit of low volume fraction, all three effective wavenumbers agree, as expected. For the largest volume fraction 40%, the expected error^2^ of k_*_ is 6%. However, the relative difference between the attenuations of k_*LS_ and k_*0_ and the multi-species attenuation of k_*_ reaches 30%.

Summarizing figures 3 and 4, all the approximations are similar for either low frequency or low volume fraction. This is because the three phases in table 2 have similar properties. In our next example one of the phases, air, will be very different from the others, which will lead to more dramatic differences.

### (b) Two-dimensional concrete

When there is a high contrast in the properties of the inclusions, multiple scattering can have a dramatic effect. To demonstrate this we consider a concrete-like material made from a limestone possessing cylinders of brick and air, given in table 3.

**Table 3.** Material properties for our concrete-like material. Note that we used compacted limestone with very low porosity [51].

	density (kg m^−3^)	speed (*m* *s*^−1^)	radius (mm)	volume %
limestone	*ρ*=2460	*c*=4855	—	84%
brick	*ρ*_*L*_=1800	*c*_*L*_=3650	*a*_*L*_=2.0	*ϕ*_*L*_=10%
dry air	*ρ*_*S*_=1.293	*c*_*S*_=331.4	*a*_*S*_=0.2	*ϕ*_*S*_=6%

**Figure 5.**
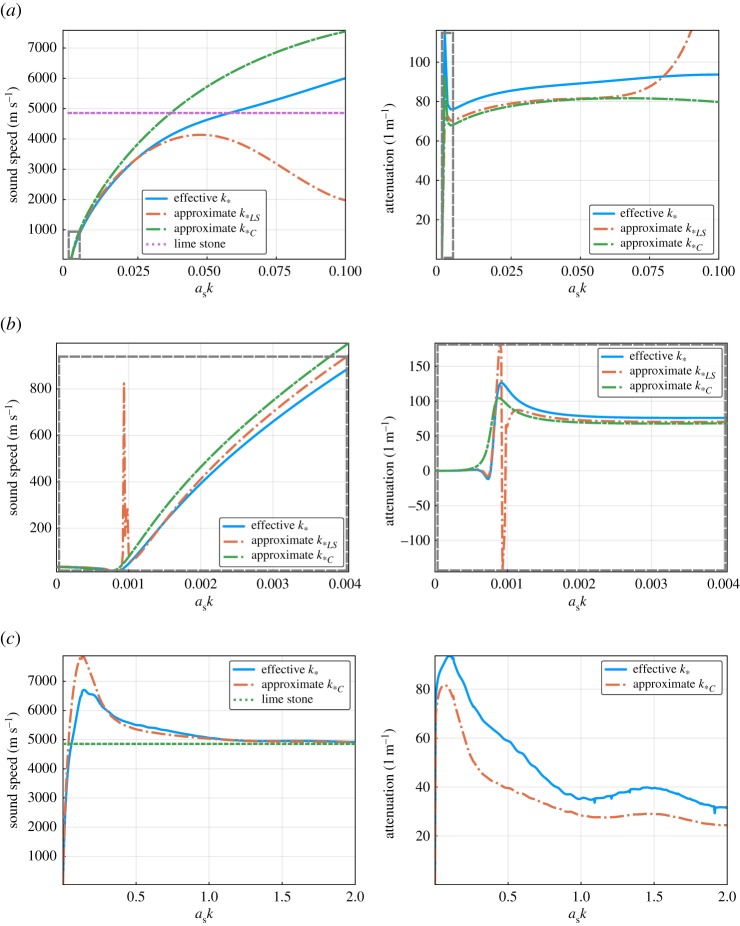
Sound speed and attenuation from the approximate wavenumbers k_*C_ (5.15) and k_*LS_ (5.7) with the more accurate k_*_ (5.10) for the concrete-like mixture shown in table 3. Code: https://github.com/arturgower/EffectiveWaves.jl/tree/master/examples/concrete. (*a*) Fixed wavenumber *k*=*k*_0_ while changing the radius *a*_*S*_, (*b*) same as (*a*) but for smaller *a*_*S*_*k* and (*c*) fixed radius *a*_*S*_ while changing the wavenumber *k*. (Online version in colour.)

Figure 5 shows that it is only in the low-frequency limit, *ka*_*S*_<0.05, that the wavenumbers k_*C_ and k_*LS_ agree with the more exact k_*_, which has a maximum expected relative error of only *ϕ*^3^=0.16^3^≈0.4%. And in figure 5*b*, the wavenumber k_*LS_ appears to hit a resonance which should not be present. This, and the dramatic changes in attenuation at low frequency, are expected because for an inclusion with low density, the effective wavenumber diverges for fixed volume fraction when *k* tends to zero [8]. Figure 5*c* shows the limitations of k_*C_ as the frequency increases. Even though k_*C_ is only valid for low frequencies, its results are quite close to k_*_, having a relative difference of around 25%.

**Figure 6.**
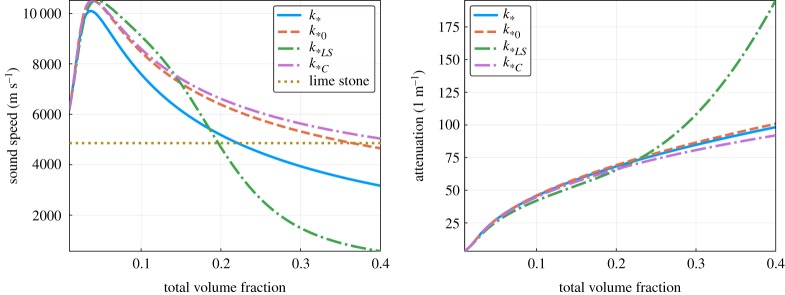
Sound speed and attenuation from the three effective wavenumbers k_*C_ (5.15), k_*0_ (5.16) and k_*LS_ (5.7), with the more accurate k_*_ (5.10), against the total volume fraction of the inclusions for the concrete shown in table 3. (Online version in colour.)

Again as expected, all the wavenumbers converge as the volume fraction decreases (figure 6), yet the differences in the wave speed are significant, reaching 100% in this example, when the total volume fraction *ϕ*=40%.

## 7. The average field and reflection

In this section, we determine the reflected field from a halfspace, which is achieved by deducing the averaged scattering coefficient $A^{n}$, for low number density shown in (4.10)_2_.

In order to calculate $A^{n}$ we first use (4.6) and (4.11), except here we can deduce that K_*0_=*k*^2^. As θ_*_ appears in (4.6), we expand

7.1 $$\theta_{*}=\theta_{*0}+\theta_{*1}n+\theta_{*2}n^{2}+O(n^{2}),$$

which combined with Snell’s equation (4.4) and the number density expansions (4.10) gives for the first two orders:

7.2 $$K_{*0}\sin{\left(\theta_{*0}\right)}^{2}=k^{2}\sin{\left(\theta_{\mathrm{in}}\right)}^{2},\;k^{2}\theta_{*1}\cos(\theta_{*0})=2i\langle \;f_{\circ }\rangle (0)\frac{\sin{\left(\theta_{*0}\right)}^{3}}{\sin{\left(\theta_{\mathrm{in}}\right)}^{2}}.$$

For $\bar{x}$, which appears in equation (4.6), we assume that, as n→0, $\bar{x}$ is a fixed width large enough for the effective wave ansatz (4.3) to hold, meaning that

7.3 $$\bar{x}=O(1),\;\int_{0}^{\bar{x}}A^{n}(x_{2},\text{s})\;e^{-i\alpha x_{2}}\;dx_{2}=O(1).$$

Using the above in (4.6) we conclude that K_*0_=*k*^2^, to ensure that $n^{-1}$ appears on the left-hand side. Subsequently, using K_*0_=*k*^2^ in (7.2) leads to

7.4 $$\theta_{*0}=\theta_{\mathrm{in}},\;\theta_{*1}=\frac{2i\langle \;f_{\circ }\rangle (0)}{k^{2}}\tan\theta_{\mathrm{in}}$$

and

7.5 $$\theta_{*2}=\theta_{*1}^{2}\left[\frac{\cos\theta_{\mathrm{in}}}{\sin\theta_{\mathrm{in}}}+\frac{1}{\sin(2\theta_{\mathrm{in}})}\right]+2i\frac{\left\langle \;f_{\circ \circ }\rangle (0\right)}{k^{2}}\tan\theta_{\mathrm{in}},$$

where θ_*2_ is given for completeness. We use the above to expand

7.6 $$\frac{e^{i(\alpha_{*}-\alpha )\bar{x}}}{\alpha_{*}-\alpha }=i\bar{x}+\left[\frac{1}{n}-\frac{\left\langle \;f_{\circ \circ }\rangle (0\right)}{\left\langle \;f_{\circ }\rangle (0\right)}\right]\frac{ik}{2\langle \;f_{\circ }\rangle (0)}\cos\theta_{\mathrm{in}}+\frac{1}{2k}\sec\theta_{\mathrm{in}}+O(n),$$

then we substitute the leading order term in the above into (4.6) leading to: $\langle \;f_{\circ }\rangle (0)=\sum_{n=-\infty }^{\infty }\int_{S}A_{*0}^{n}\;d{\text{s}}_{2}^{n}$. However, from (B.17) we found that $A_{*0}^{n}$ is independent of *n* and **s**_2_, therefore, $A_{*0}^{n}=-1$. This means that $A_{*}^{n}$ tends, in the limit n→0, to the scattering coefficient of one lone cylinder:

7.7 $$A^{m}({\text{x}}_{1},{\text{s}}_{1})\;\to \;i^{m}A_{*0}^{m}({\text{s}}_{1})\;e^{-im\theta_{*0}}\;e^{i{\text{x}}_{1}\cdot \sqrt{{\text{K}}_{*0}}}=-i^{m}\;e^{-im\theta_{\mathrm{in}}}\;e^{i{\text{x}}_{1}\cdot \text{k}}=A_{1}^{m},$$

where we used K_*0_=*k*^2^ and θ_*0_=*θ*_in_, and the last equation is from (4.15).

To calculate the next order in n of equation (4.6) we need to calculate $b(\bar{x})$. To do so, we assume that $A^{n}({\text{x}}_{2},{\text{s}}_{2})\to A_{2}^{n}$ as n→0 for every *x*_2_>0. That is, in the limit where there are no cylinders, except one fixed at **x**_2_, the averaged scattering coefficient $A^{n}$ tends to the scattering coefficient of one lone cylinder, even for $0<x_{2}<\bar{x}$. As a result

$$A^{n}(x_{2},0,{\text{s}}_{2})=-i^{n}\;e^{-in\theta_{\mathrm{in}}}\;e^{i\alpha x_{2}}+O(n),\;\text{for}\;x_{2}>0,$$

which when substituted in $b(\bar{x})$ from (4.6), together with (4.12), leads to $b(\bar{x})=i\bar{x}\langle \;f_{\circ }\rangle (0)+O(n)$. Substituting this, (7.6), and (7.5) into (4.6), and then ignoring second-order terms $O(n^{2})$ we obtain

7.8 $$\frac{-i\langle \;f_{\circ \circ }\rangle (0)k\cos\theta_{\mathrm{in}}}{2\langle \;f_{\circ }\rangle (0)}+\frac{ik\cos\theta_{\mathrm{in}}}{2\langle \;f_{\circ }\rangle (0)}\sum_{n=-\infty }^{\infty }\int_{S}A_{*1}^{n}({\text{s}}_{2})\;d{\text{s}}_{2}^{n}=-\frac{\left\langle \;f_{\circ }\rangle (0\right)}{2k\cos\theta_{\mathrm{in}}}+O(n^{2}),$$

where we also used $\sum_{n=-\infty }^{\infty }\int_{S}n\;d{\text{s}}_{2}^{n}=0,$ which is a result of the property $Z_{2}^{n}=Z_{2}^{-n}$, see (2.5), implying that $d{\text{s}}_{2}^{n}=d{\text{s}}_{2}^{-n}$. In appendix Bb, we showed that the quantity F_*_, given by (B.20), is independent of *n* and **s**_2_. So if we substitute $A_{*1}^{n}({\text{s}}_{2})$ for F_*_, we can then take F_*_ outside the sum and integral in (7.8), and then substitute back $A_{*1}^{n}({\text{s}}_{2})$ to arrive at

7.9 $$A_{*1}^{n}({\text{s}}_{2})=-\frac{i\langle \;f_{\circ }\rangle (0)}{k^{2}\cos^{2}\theta_{\mathrm{in}}}-\pi \sum_{m=-\infty }^{\infty }\int_{S}a_{12}^{2}d_{m-n}(ka_{12})\;d{\text{s}}_{1}^{m}+O(n^{2}),$$

where we used (4.12)_2_. The above reduces to the one-species case given in ([52], eqn (27)). With $A_{*1}^{n}$ and $A_{*0}^{n}$, we can now calculate reflection from a halfspace.

### (a) Reflection from a halfspace

Here we calculate the reflected wave measured at (*x*,*y*), where *x*<0. To achieve this, we assume that the boundary layer around *x*=0 has little effect on the reflected wave, that is, we assume most of the scatterers behave as if they are in an infinite medium. This is the same as taking $\bar{x}=0$, which was also used in [52], where they showed that this approach matches other homogenization results in the low-frequency limit. We note, however, that Felbacq *et al.* [53] discuss the possibility of a boundary layer effect even in the low-frequency limit.

Substituting (3.9) into the total effective wave (3.8), and using the form (4.3) reveals

7.10 $$\langle u(x,y)\rangle =e^{ik\cdot x}+n\;e^{-im\theta_{*}}\sum_{m=-\infty }^{\infty }i^{m}\int_{S}A_{*}^{m}({\text{s}}_{1})\int_{0<x_{1}<\infty }e^{i\beta y_{1}+i\alpha_{*}x_{1}}\Phi_{m}(kR_{1},\Theta_{1})\;dx_{1}\;d{\text{s}}_{1}^{m},$$

where we used *u*_in_(*x*,*y*)=*e*^*i****k***⋅***x***^, $d{\text{s}}_{1}^{m}=Z_{1}^{m}p({\text{s}}_{1})\;d{\text{s}}_{1}$, $\Phi_{m}(kR_{1},\Theta_{1})=H_{m}^{\left(1\right)}(kR_{1})\;e^{im\Theta_{1}}$, substituted (3.14), used $N=|R_{N}|n$ and took the limit N→∞. Then using (B.9) and (B.11), we obtain

7.11 $$\int_{0<x_{1}<\infty }e^{i\beta y_{1}+i\alpha_{*}x_{1}}\Phi_{m}(kR_{1},\Theta_{1})\;dx_{1}=e^{-i\alpha x+i\beta y}\frac{2}{\alpha }\frac{{\left(-i\right)}^{-m}i}{\alpha +\alpha_{*}}\;e^{-im\theta_{\mathrm{in}}},$$

noting that *x*_1_−*x*>0. Using the above in (7.10), we reach

**Figure 7.**
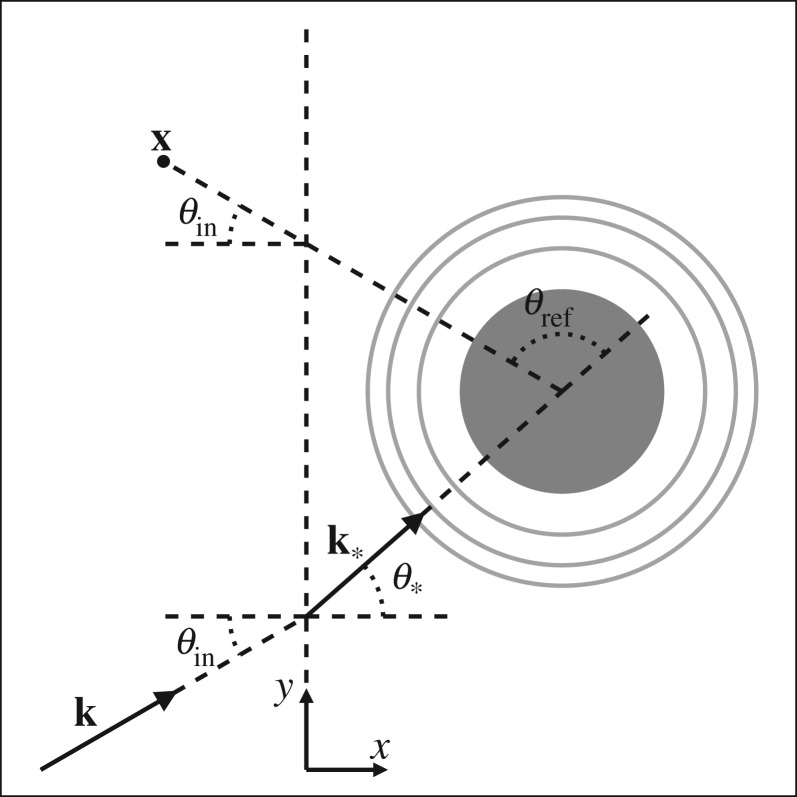
The far-field reflected angle *θ*_ref_, where **k**=(*α*,*β*) and k_*_ is the effective transmitted wavenumber defined in §4. The wavenumber k_*_ results from ensemble averaging all scattered waves originating from *x*>0 (to the right of the dashed vertical line). The reflected field measured at **x** can be understood as the scattering (the grey circles) of the transmitted wave by an effective particle (grey particle). In the figure, *θ*_ref_ equals *π*−θ_*_−*θ*_in_, but for small number density $\theta_{*}=\theta_{\mathrm{in}}+O(n)$, which is why *θ*_ref_=*π*−2*θ*_in_ appears in (1.3) and (1.4).

7.12 $$\langle u(x,y)\rangle =e^{ik\cdot x}+\frac{2n}{\alpha }\frac{i\;e^{-i\alpha x+i\beta y}}{\alpha +\alpha_{*}}\sum_{m=-\infty }^{\infty }e^{im\theta_{\mathrm{ref}}}\int_{S}A_{*}^{m}({\text{s}}_{1})\;d{\text{s}}_{1}^{m},$$

where *θ*_ref_=*π*−θ_*_−*θ*_in_. The reflected wave shown by (1.2) is calculated by expanding for small n, including $\theta_{\mathrm{ref}}=\pi -2\theta_{\mathrm{in}}+O(n)$, and then substituting the results from §7. For a single-species this formula reduces to ([52], eqn (41) and (42)). Figure 7 gives a pictorial representation of the reflection coefficient in (7.12).

## 8. Conclusion

We have deduced the effective wavenumbers (1.1) and (1.5), and reflection coefficient (1.2), for a multi-species material up to moderate number density and over a broad range of frequencies. This will enable experimental researchers to extract more information about the makeup of inhomogeneous media (see the electronic supplementary material for self-contained expressions for the wavenumbers and reflection coefficients in the case of a finite number of species). We also remark that the results may be extended straightforwardly to multiple scattering from cylinders in a number of contexts, including two-dimensional electromagnetism.

Characterization is not the only application; this theory can also be employed to design novel materials. We have shown that multiple scattering between different species can lead to effective properties that are not exhibited by single-species media. That is, using our multi-species formulae it is now possible to choose species so as to design impedance matched, highly dispersive and broad band attenuating materials.

We also saw that the multi-species effective wavenumbers derived in the acoustics literature were accurate for low frequency and low volume fraction. But to go beyond these limitations, our more precise effective wavenumber was needed. We also illustrated that a ‘self-consistent’ approach to calculating the effective wavenumber is not even accurate at low frequencies.

Two main issues of our method deserve further investigation: the effects of the boundary layer near the surface of the halfspace and the QCA. To calculate the reflection coefficient up to second order in the number density, we neglected the effects of the boundary layer. It is not clear how to theoretically make progress without these two approximations, nor what errors they introduce. We believe that these issues represent important future work.

## Supplementary Material

Notes on effective waves in a multi-species material

## Footnotes

1 In this case, we did not exactly use the two-dimensional version of eqn (23) from [28], but instead used a more accurate version where we summed enough terms for the far-field patterns to converge.

2 If we disregard the error due to the low-frequency assumptions, we can estimate the expected errors *ϕ*^2^=0.4^2^=16% for *k*_*0_ and 2*ϕ*_*L*_*ϕ*_*S*_=2*0.2^2^=8% for both *k*_**LS*_ and *k*_**C*_.

## Data accessibility

We provide the code to generate all the graphs in [31]. We also provide electronic supplementary material with self-contained formulae.

## Appendix A. Effective wavenumber for multi-species spherical inclusions

In this section, we apply our multi-species theory to the results in [21] for spherical inclusions to reach the effective wavenumber (1.5). Details are omitted when the results follow by direct analogy. For spheres we define the ensemble-average far-field pattern and multiple scattering pattern,

A 1 $$\langle F_{\circ }\rangle (\theta )=-\sum_{n=0}^{\infty }P_{n}(\cos\theta )\int_{S}(2n+1)\;d{\text{s}}_{1}^{n}$$

and

A 2 $$\begin{array}{rl} \left\langle F_{\circ \circ }\right\rangle & \;=\frac{i{\left(4\pi \right)}^{2}}{2}\sum_{n=0}^{\infty }\sum_{p=0}^{\infty }\sum_{q}\int_{S}\int_{S}\frac{\sqrt{\left(2n+1)(2p+1\right)}}{{\left(4\pi \right)}^{3/2}}\\ & \;\sqrt{2q+1}G(n,0;p,0;q)ka_{12}D_{q}(ka_{12})\;d{\text{s}}_{1}^{n}\;d{\text{s}}_{2}^{p}, \end{array}$$

where *q* takes the values

|n−p|,|n−p|+2,|n−p|+4,…,n+p,

$d{\text{s}}_{i}^{n}=\zeta^{n}({\text{s}}_{i})p({\text{s}}_{i})\;d{\text{s}}_{i}$, *D*_*m*_(*x*)=*xj*_*m*_′(*x*)(*xh*_*m*_′(*x*)+*h*_*m*_(*x*))+(*x*^2^−*m*(*m*+1))*j*_*m*_(*x*)*j*_*m*_(*x*), *P*_*n*_ are Legendre polynomials, *j*_*m*_ are spherical Bessel functions, *h*_*m*_ are spherical Hankel functions of the first kind and

A 3 $$\zeta^{m}({\text{s}}_{j})=\frac{q_{j}j_{m}^{\prime }(ka_{j})j_{m}(k_{j}a_{j})-j_{m}(ka_{j})j_{m}^{\prime }(k_{j}a_{j})}{q_{j}h_{m}^{\prime }(ka_{j})j_{m}(k_{j}a_{j})-h_{m}(ka_{j})j_{m}^{\prime }(k_{j}a_{j})}=\zeta^{-m}({\text{s}}_{j}),$$

with *q*_*j*_=(*ρ*_*j*_*c*_*j*_)/(*ρc*), where G is a Gaunt coefficient ([21], eqn (A.5)) defined here as

A 4 $$G(n,0;p,0;q)=\frac{\sqrt{\left(2n+1)(2p+1)(2q+1\right)}}{2\sqrt{4\pi }}\int_{0}^{\pi }P_{n}(\cos\theta )P_{p}(\cos\theta )P_{q}(\cos\theta )\sin\theta \;d\theta .$$

## Appendix B. Calculating the effective equations (4.6) and (4.5)

In this appendix, we provide an in-depth outline of the derivation for (4.6) and (4.5), which are given in terms of the unknowns $A^{n}({\text{x}}_{2},\text{s})$ and *k*_*_. The approach here is similar to Section IV in [5].

We begin by substituting (4.1) and (4.3) into the integral in the governing equation (3.24) which for $x_{1}>\bar{x}+a_{21}$ takes the form

B 1 $$\begin{array}{rl} & \;\int_{\overset{x_{2}>0}{R_{21}>a_{21}}}A^{n}({\text{x}}_{2},\text{s})\Phi_{n-m}(kR_{21},\Theta_{21})\;d{\text{x}}_{2}\\ & \;\;=e^{i\beta y_{1}}\;e^{-i(n-m)\pi }\int_{0}^{\bar{x}}A^{n}(x_{2},0,\text{s})L_{n-m}(x_{2}-x_{1})\;dx_{2}\\ & \;\;+i^{n}\;e^{-in\theta_{*}}\;e^{-i(n-m)\pi }A_{*}^{n}\;e^{i{\text{k}}_{*}\cdot {\text{x}}_{1}}\int_{\overset{X>\bar{x}-x_{1}}{R>a_{21}}}\;e^{i{\text{k}}_{*}\cdot \text{X}}\Phi_{n-m}(kR,\Theta )\;d\text{X}, \end{array}$$

where *X*=*x*_2_−*x*_1_ and *Y* =*y*_2_−*y*_1_, so that (*R*,*Θ*) are the polar coordinates of **X**=(*X*,*Y*), where *R*=*R*_21_ and *Θ*=*Θ*_21_−*π*, and we define

B 2 $$\Phi_{n-m}(kR,\Theta ):=e^{i(n-m)\Theta }H_{n-m}(kR),\;L_{n-m}(X):=\int_{-\infty }^{\infty }e^{i\beta Y}\Phi_{n-m}(kR,\Theta )\;dY.$$

From eqn (37) of [52], we have

B 3 $$L_{n}(X)=\left\{ \begin{array}{ll} \frac{2}{\alpha }{\left(-i\right)}^{n}\;e^{-in\theta_{\mathrm{in}}}\;e^{i\alpha X}, & \text{for}\;X>0,\\ \frac{2}{\alpha }i^{n}\;e^{in\theta_{\mathrm{in}}}\;e^{-i\alpha X}, & \text{for}\;X<0. \end{array}\right.$$

To calculate the last integral in (B 1), it is necessary that *k*_*_≠*k*, as *k*_*_=*k* leads to a divergent integrand. Assuming *k*_*_≠*k*, we observe that

$$e^{i{\text{k}}_{*}\cdot \text{X}}\Phi_{n-m}(kR,\Theta )=\frac{1}{k^{2}-k_{*}^{2}}[\Phi_{n-m}(kR,\Theta )\nabla^{2}\;e^{i{\text{k}}_{*}\cdot \text{X}}-e^{i{\text{k}}_{*}\cdot \text{X}}\nabla^{2}\Phi_{n-m}(kR,\Theta )]$$

because ∇^2^*Φ*_*n*−*m*_(*kR*,*Θ*)=−*k*^2^*Φ*_*n*−*m*_(*kR*,*Θ*) and $\nabla^{2}\;e^{i{\text{k}}_{*}\cdot \text{X}}=-k_{*}^{2}\;e^{i{\text{k}}_{*}\cdot \text{X}}$. Then by Green’s second identity, we obtain

B 4 $$\begin{array}{rl} & \;\int_{\overset{X>\bar{x}-x_{1}}{R>a_{21}}}[\Phi_{n-m}(kR,\Theta )\nabla^{2}\;e^{i{\text{k}}_{*}\cdot \text{X}}-e^{i{\text{k}}_{*}\cdot \text{X}}\nabla^{2}\Phi_{n-m}(kR,\Theta )]\;d\text{X}\\ & \;\;=\int_{\partial B}\left[\Phi_{n-m}(kR,\Theta )\frac{\partial \;e^{i{\text{k}}_{*}\cdot \text{X}}}{\partial \text{n}}-e^{i{\text{k}}_{*}\cdot \text{X}}\frac{\partial \Phi_{n-m}(kR,\Theta )}{\partial \text{n}}\right]ds, \end{array}$$

where **n** is the outwards pointing unit normal. For *x*_1_>*a*_21_, the region B is given by $X>\bar{x}-x_{1}$ and *R*>*a*_21_, so that the boundary ∂B is the circle *R*=*a*_21_ and the line $X=\bar{x}-x_{1}$. The integral on the left-hand side of (B 4) reduces to the form

B 5 $$\int_{B}e^{i{\text{k}}_{*}\cdot \text{X}}\Phi_{n-m}(kR,\Theta )\;d\text{X}=\frac{1}{\alpha^{2}-\alpha_{*}^{2}}(M_{\circ }+M_{-}),$$

where $k^{2}-k_{*}^{2}=\alpha^{2}-\alpha_{*}^{2}$. Here, we have [5], eqn (68):

B 6 $$M_{\circ }=2\pi i^{n-m}\;e^{i(n-m)\theta_{*}}N_{n-m}(ka_{12},k_{*}a_{12}),$$

where $N_{n-m}$ is defined by (4.8), and

B 7 $$\begin{array}{rl} M_{-} & \;=-\int_{-\infty }^{\infty }\left[\Phi_{n-m}(kR,\Theta )\frac{\partial \;e^{i{\text{k}}_{*}\cdot \text{X}}}{\partial X}-e^{i{\text{k}}_{*}\cdot \text{X}}\frac{\partial \Phi_{n-m}(kR,\Theta )}{\partial X}\right]dY\\ & \;=\left\{ \begin{array}{ll} 2\frac{\alpha_{*}+\alpha }{\alpha }i^{n-m-1}\;e^{i(\alpha_{*}-\alpha )X}\;e^{i(n-m)\theta_{\mathrm{in}}}, & \text{for}\;X<0,\\ 2\frac{\alpha_{*}-\alpha }{\alpha }i^{-(n-m)-1}\;e^{i(\alpha_{*}+\alpha )X}\;e^{-i(n-m)\theta_{\mathrm{in}}}, & \text{for}\;X>0, \end{array}\right. \end{array}$$

for $X=\bar{x}-x_{1}<0$, which is identical to ([5], eqn (67)) (with the replacements $\ell \mapsto \bar{x}$ and *λ*↦*α*_*_, and where we have included the case *X*>0 for future reference).

From (B 5), it follows that (B 1) now becomes

$$\int_{\overset{x_{2}>0}{R_{21}>a_{21}}}A^{n}({\text{x}}_{2},\text{s})\Phi_{n-m}(kR_{21},\Theta_{21})\;d{\text{x}}_{2}=i^{m}\;e^{i\beta y_{1}}[e^{-im\theta_{*}}\;e^{i\alpha_{*}x_{1}}B^{n,m}+e^{i\alpha x_{1}}\;e^{i(n-m)\theta_{\mathrm{in}}}C^{n,m}],$$

where

B 8 $$B^{n,m}=A_{*}^{n}\frac{2\pi }{k^{2}-k_{*}^{2}}N_{n-m}(ka_{12},k_{*}a_{12})$$

and

B 9 $$C^{n,m}=\frac{2}{\alpha }\int_{0}^{\bar{x}}{\left(-i\right)}^{n}A^{n}(x_{2},0,\text{s})\;e^{-i\alpha x_{2}}\;dx_{2}+\frac{2}{\alpha }iA_{*}^{n}\;e^{-in\theta_{*}}\frac{e^{i(\alpha_{*}-\alpha )\bar{x}}}{\alpha_{*}-\alpha }.$$

Substituting the above into (3.24), assuming $x_{1}>\bar{x}+a_{21}$, and cancelling common factors, we arrive at

B 10 $$\begin{array}{r} e^{i\alpha_{*}x_{1}}\;e^{-im\theta_{*}}\left[A_{*}^{m}+n\sum_{n=-\infty }^{\infty }\int_{S}B^{n,m}\;d{\text{s}}_{2}^{n}\right]+e^{i\alpha x_{1}}\;e^{-im\theta_{\mathrm{in}}}\left[n\sum_{n=-\infty }^{\infty }e^{in\theta_{\mathrm{in}}}\int_{S}C^{n,m}d{\text{s}}_{2}^{n}+1\right]=0, \end{array}$$

having also used *I*_1_=*e*^*i*(*α*_*_*x*_1_+*βy*_1_)^. As the above must hold for all *x*_1_, we can equate the terms in the brackets to zero, which leads to (4.6) and (4.5).

**(a) Effective wave for any pair distribution**

To calculate the effective wave for any pair distribution function *χ* satisfying (3.21), we substitute (3.19) into (3.24), which leads to an extra integral appearing on the left side of (B 1):

$$\begin{array}{rl} & \;\int_{\overset{R_{\infty }}{R_{21}>a_{21}}}A^{n}({\text{x}}_{2},{\text{s}}_{2})\Phi_{n-m}(kR_{21},\Theta_{21})\chi (R_{21}|{\text{s}}_{1},{\text{s}}_{2})\;d{\text{x}}_{2}\\ & \;\;=i^{n}A_{*}^{n}({\text{s}}_{2})\;e^{-in\theta_{*}}\;e^{-i(n-m)\pi }\;e^{i{\text{k}}_{*}\cdot {\text{x}}_{1}}\int_{\overset{X>\bar{x}-x_{1}}{a_{21}<R<{\bar{a}}_{21}}}\;e^{i{\text{k}}_{*}\cdot \text{X}}\Phi_{n-m}(kR,\Theta )\chi (R|{\text{s}}_{1},{\text{s}}_{2})\;d\text{X}\\ & \;\;+e^{-i(n-m)\pi }\int_{\overset{-x_{1}\leq X\leq \bar{x}-x_{1}}{a_{21}<R<{\bar{a}}_{21}}}A^{n}({\text{x}}_{2},{\text{s}}_{2})\Phi_{n-m}(kR,\Theta )\chi (R|{\text{s}}_{1},{\text{s}}_{2})\;d{\text{x}}_{2}, \end{array}$$

where *X*=*x*_2_−*x*_1_ and *Y* =*y*_2_−*y*_1_, so that (*R*,*Θ*) are the polar coordinates of **X**=(*X*,*Y*), where *R*=*R*_21_, *Θ*=*Θ*_21_−*π*. The second integral on the right is zero when $x_{1}>\bar{x}+{\bar{a}}_{21}$ because then

$$-x_{1}\leq X\leq \bar{x}-x_{1}<-{\bar{a}}_{21}$$

and **X** can not satisfy both $X<-{\bar{a}}_{21}$ and $a_{21}<R<{\bar{a}}_{21}$.

For $x_{1}>\bar{x}+{\bar{a}}_{21}$, the integral over the regions $X>\bar{x}-x_{1}$ and $a_{21}<R<{\bar{a}}_{21}$ reduces to the just $a_{21}<R<{\bar{a}}_{21}$, so leaving out the factors on the left, and using $e^{i{\text{k}}_{*}\cdot \text{X}}=e^{ik_{*}R\cos(\Theta -\theta_{*})}$, the integral becomes

B 11 $$\begin{array}{rl} & \;\int_{a_{21}<R<{\bar{a}}_{21}}\int_{0}^{2\pi }e^{ik_{*}R\cos(\Theta -\theta_{*})}\;e^{i(n-m)\Theta }H_{n-m}(kR)\chi (R\;|\;{\text{s}}_{1},{\text{s}}_{2})R\;d\Theta \;dR,\\ & \;\;=2\pi i^{n-m}\;e^{i(n-m)\theta_{*}}X_{*}, \end{array}$$

where we used the Jacobi–Anger expansion and $X_{*}$ is defined by (4.9). In conclusion, we should sum $2\pi A_{*}^{n}({\text{s}}_{2})X_{*}$ to $B_{n,m}$, appearing in equations (B 8) and (B 10).

**(b) Low number fraction**

To calculate *k*^2^_*_ we need only equation (4.5), which after substituting (4.10) and

B 12 $$N_{m}(ka_{12},k_{*}a_{12})∼\frac{2i}{\pi }+nK_{*1}\frac{a_{21^{2}}}{2}d_{m}(ka_{21}),$$

with *d*_*m*_ defined by (4.14), and then equating terms of order O(1) and O(n), leads to

B 13 $$A_{*0}^{m}({\text{s}}_{1})=\frac{4i}{K_{*1}}\sum_{n=-\infty }^{\infty }\int_{S}A_{*0}^{n}({\text{s}}_{2})\;d{\text{s}}_{2}^{n}$$

and

B 14 $$\begin{array}{rl} & A_{*1}^{m}({\text{s}}_{1})-\pi \sum_{n=-\infty }^{\infty }\int_{S}a_{21}^{2}d_{n-m}(ka_{21})A_{*0}^{n}({\text{s}}_{2})\;d{\text{s}}_{2}^{n}\\ & \;\;=\frac{4i}{K_{*1}}\sum_{n=-\infty }^{\infty }\int_{S}\left[A_{*1}^{n}({\text{s}}_{2})-A_{*0}^{n}({\text{s}}_{2})\frac{K_{*2}}{K_{*1}}\right]d{\text{s}}_{2}^{n}. \end{array}$$

Turning to (B 13), we see that $A_{*0}^{m}({\text{s}}_{1})$ is independent of both *m* and **s**_1_. Let $A_{*0}:=A_{*0}^{n}({\text{s}}_{2})=A_{*0}^{m}({\text{s}}_{1})$, then we can divide both sides of (B 13) by $A_{*0}$ (assuming $A_{*0}\neq 0$), and use (4.12) to arrive at

B 15 $$K_{*1}=-4i\langle \;f_{\circ }\rangle (0).$$

Turning to (B 14), we see that

B 16 $$F_{*}=A_{*1}^{m}({\text{s}}_{1})-\pi A_{*0}\sum_{n=-\infty }^{\infty }\int_{S}a_{21}^{2}\;d_{n-m}(ka_{21})\;d{\text{s}}_{2}^{n}$$

is independent of both *m* and **s**_1_, which we use to write (B 14) as

$$\begin{array}{rl} F_{*} & \;=-\frac{1}{\left\langle \;f_{\circ }\rangle (0\right)}\sum_{n=-\infty }^{\infty }\int_{S}A_{*1}^{n}({\text{s}}_{2})\;d{\text{s}}_{2}^{n}-iA_{*0}\frac{K_{*2}}{4\langle \;f_{\circ }\rangle (0)}\\ & \;=-\frac{F_{*}}{\left\langle \;f_{\circ }\rangle (0\right)}\sum_{n=-\infty }^{\infty }\int_{S}d{\text{s}}_{2}^{n}-iA_{*0}\frac{K_{*2}}{4\langle \;f_{\circ }\rangle (0)}+A_{*0}\frac{\left\langle \;f_{\circ \circ }\rangle (0\right)}{\left\langle \;f_{\circ }\rangle (0\right)}, \end{array}$$

where we used (4.13). This simplifies to *K*_*2_=−4*i*〈 *f*_°°_〉(0), which together with (B 15), (4.10) leads to the effective wavenumber (4.11).
