# Supplementary material for: Reflection from a multi-species material and its transmitted effective wavenumber
Source: Proc Math Phys Eng Sci. 2018 Apr 11;474(2212):20170864. doi: 10.1098/rspa.2017.0864 (PMC5938676; doi:10.1098/rspa.2017.0864)
Supplement: Notes on effective waves in a multi-species material [file rspa20170864supp1.pdf]

Notes on effective waves in a multi-species material

Artur L. Gower[†], Michael J. A. Smith[†], William J. Parnell[†], and I. David Abrahams[⋆]

[†]School of Mathematics, University of Manchester, Oxford Road, Manchester M13 9PL, UK

[⋆]Isaac Newton Institute for Mathematical Sciences, 20 Clarkson Road, Cambridge CB3 0EH, UK

March 6, 2018

Abstract

This Supplementary Material is a self-contained document providing further detail on the calculation of effective wavenumbers for uniformly distributed multi-species inclusions. The formulae for multi-species cylinders and spheres are given here, in addition to expressions describing reflection from a halfspace filled with cylinders. Code to implement the formulas is given in `https://github.com/arturgower/EffectiveWaves.jl`. For detailed derivations see our paper "Reflection from a multi-species material and its transmitted effective wavenumber", which shows how to introduce a pair-correlation between the species.

Keywords: polydisperse, multiple scattering, multi-species, effective waves, quasicrystalline approximation, statistical methods

1 Effective waves for uniformly distributed species

We consider a halfspace $x > 0$ filled with S types of inclusions (species) that are uniformly distributed. The fields are governed by the scalar wave equation:

$$\nabla^2 u + k^2 u = 0, \quad \text{(in the background material)} \tag{1}$$

$$\nabla^2 u + k_j^2 u = 0, \quad \text{(inside the } j\text{-th scatterer)}, \tag{2}$$

The background and species material properties are summarised in Table 1. The goal is to find an effective homogeneous medium with wavenumber k_*, where waves propagate, in an ensemble average sense, with the same speed and attenuation as they would in a material filled with scatterers. See Gower (2017) for the code that implements the formulas below.

Below we present the effective wavenumber, for any incident wavenumber and moderate number fraction, when the species are either all cylinders or spheres[*]. For cylindrical inclusions we also present the reflection of a plane wave from this multi-species material.

Background properties:	wavenumber k	density ρ	sound speed c	
Specie properties:	number density $\mathfrak{n}_j$	density ρ_j	sound speed c_j	radius a_j
total number density $\mathfrak{n}$	effective wavenumber k_*	species min. distance $a_{j\ell} > a_j + a_\ell$		

Table 1: Summary of material properties and notation. The index j refers to properties of the j-th species. Note a typical choice for $a_{j\ell}$ is $a_{j\ell} = c(a_j + a_\ell)$, where $c = 1.01$.

[*]In principal these formulas can be extended to include different shaped scatterers by using Waterman's T-matrix Waterman (1971); Varadan et al. (1978); Mishchenko et al. (1996)

1.1 Cylindrical species

We consider an incident wave

$$\mathrm{e}^{\mathrm{i}\mathbf{k}\cdot\mathbf{x}} \quad \text{with} \quad \mathbf{k}\cdot\mathbf{x} = kx\cos\theta_{\text{in}} + ky\sin\theta_{\text{in}}, \tag{3}$$

and angle of incidence θ_{in} from the x-axis, exciting a material occupying the halfspace $x > 0$. Then, assuming low number density $\mathfrak{n}$ (or low volume fraction $\sum_\ell \pi a_\ell^2 \mathfrak{n}_\ell$), the effective transmitted wavenumber k_* becomes

$$k_*^2 = k^2 - 4\mathrm{i}\mathfrak{n}\langle f_\circ\rangle(0) - 4\mathrm{i}\mathfrak{n}^2\langle f_{\circ\circ}\rangle(0) + \mathcal{O}(\mathfrak{n}^3), \tag{4}$$

with $\langle f_\circ\rangle$ and $\langle f_{\circ\circ}\rangle$ given by (8). The above reduces to Linton and Martin (2005) equation (81) for a single species in the low frequency limit; This equation (81) has been confirmed by several independent methods Martin et al. (2010); Martin and Maurel (2008); Chekroun et al. (2012); Kim (2010).

The ensemble-average reflected wave measured at $x < 0$ is given by

$$\langle u_{\text{ref}}\rangle = \frac{\mathfrak{n}}{\alpha^2}\left[R_1 + \mathfrak{n}R_2\right]\mathrm{e}^{-\mathrm{i}\alpha x + \mathrm{i}\beta y} + \mathcal{O}(\mathfrak{n}^3), \tag{5}$$

where

$$R_1 = \mathrm{i}\langle f_\circ\rangle(\theta_{\text{ref}}), \quad \theta_{\text{ref}} = \pi - 2\theta_{\text{in}}, \tag{6}$$

$$R_2 = \frac{2\langle f_\circ\rangle(0)}{k^2\cos^2\theta_{\text{in}}}\left[\sin\theta_{\text{in}}\cos\theta_{\text{in}}\langle f_\circ\rangle'(\theta_{\text{ref}}) - \langle f_\circ\rangle(\theta_{\text{ref}})\right] + \mathrm{i}\langle f_{\circ\circ}\rangle(\theta_{\text{ref}}), \tag{7}$$

which reduces to Martin (2011) equations (40-41) for a single species, which they show agrees with other known results for small k.

The ensemble-average far-field pattern and multiple-scattering pattern are[†]

$$\langle f_\circ \rangle(\theta) = -\sum_{\ell=1}^{S} \sum_{n=-\infty}^{\infty} \frac{\mathfrak{n}_\ell}{\mathfrak{n}} Z_\ell^n \mathrm{e}^{\mathrm{i}n\theta},$$

$$\langle f_{\circ\circ} \rangle(\theta) = -\pi \sum_{\ell,j=1}^{S} \sum_{m,n=-\infty}^{\infty} a_{\ell j}^2 d_{n-m}(k a_{\ell j}) \frac{\mathfrak{n}_\ell \mathfrak{n}_j}{\mathfrak{n}^2} Z_\ell^n Z_j^m \mathrm{e}^{\mathrm{i}n\theta}, \tag{8}$$

where $d_m(x) = J_m'(x) H_m'(x) + (1 - (m/x)^2) J_m(x) H_m(x)$, the J_m are Bessel functions, the H_m are Hankel functions of the first kind and $a_{\ell j} > a_\ell + a_j$ is some fixed distance. The Z_j^m describe the type of scatterer:

$$Z_j^m = \frac{q_j J_m'(k a_j) J_m(k_j a_j) - J_m(k a_j) J_m'(k_j a_j)}{q_j H_m'(k a_j) J_m(k_j a_j) - H_m(k a_j) J_m'(k_j a_j)} = Z_j^{-m}, \tag{9}$$

with $q = (\rho_j c_j)/(\rho c)$. For instance, taking the limits $q \to 0$ or $q \to \infty$, recovers Dirichlet or Neumann boundary conditions, respectively.

1.2 Effective wavenumber for multi-species spheres

The results here are derived by applying the theory in our paper to the results in Linton and Martin (2006). We omit the details as the result follows by direct analogy.

For spherical inclusions the transmitted wavenumber becomes,

$$k_*^2 = k^2 - \mathfrak{n} \frac{4\pi \mathrm{i}}{k} \langle F_\circ \rangle(0) + \mathfrak{n}^2 \frac{(4\pi)^2}{k^4} \langle F_{\circ\circ} \rangle + \mathcal{O}(\mathfrak{n}^3), \tag{10}$$

where for spheres we define the ensemble-average far-field pattern and multiple-scattering

[†]Note we introduced the terminology "multiple-scattering pattern".

pattern,

$$\langle F_\circ \rangle(\theta) = -\sum_{n=0}^{\infty} P_n(\cos\theta) \sum_{j=1}^{S} (2n+1)\zeta_j^n \frac{\mathfrak{n}_j}{\mathfrak{n}}, \tag{11}$$

$$\langle F_{\circ\circ} \rangle = \frac{\mathrm{i}(4\pi)^2}{2} \sum_{n,p=0}^{\infty} \sum_{j,\ell=1}^{S} \sum_{q} \frac{\sqrt{(2n+1)(2p+1)}}{(4\pi)^{3/2}} \sqrt{2q+1}\mathcal{G}(n,p,q) k a_{j\ell} D_q(k a_{j\ell}) \zeta_j^n \zeta_\ell^p \frac{\mathfrak{n}_j \mathfrak{n}_\ell}{\mathfrak{n}^2},$$

where

$$D_m(x) = x j_m'(x)(x h_m'(x) + h_m(x)) + (x^2 - m(m+1)) j_m(x) j_m(x),$$

P_n are Legendre polynomials, j_m are spherical Bessel functions, h_m are spherical Hankel functions of the first kind and

$$\zeta_j^m = \frac{q_j j_m'(k a_j) j_m(k_j a_j) - j_m(k a_j) j_m'(k_j a_j)}{q_j h_m'(k a_j) j_m(k_j a_j) - h_m(k a_j) j_m'(k_j a_j)} = \zeta_j^{-m}, \tag{12}$$

with $q = (\rho_j c_j)/(\rho c)$, where the $\mathcal{G}$ is a Gaunt coefficient and is equal to

$$\mathcal{G}(n,p,q) = \frac{\sqrt{(2n+1)(2p+1)(2q+1)}}{2\sqrt{4\pi}} \int_0^{\pi} P_n(\cos\theta) P_p(\cos\theta) P_q(\cos\theta) \sin\theta d\theta.$$

See Caleap et al. (2012) for details on reflection from a single species, although, to our knowledge, a formula for reflection from a single species valid for moderate number fraction and any wavenumber has not yet been deduced.

References

Caleap, M., B. W. Drinkwater, and P. D. Wilcox (2012). "Effective dynamic constitutive parameters of acoustic metamaterials with random microstructure". In: *New Journal*

of Physics 14(3), p. 033014. DOI: 10.1088/1367-2630/14/3/033014. URL: http://stacks.iop.org/1367-2630/14/i=3/a=033014.

Chekroun, M., L. Le Marrec, B. Lombard, and J. Piraux (2012). "Time-domain numerical simulations of multiple scattering to extract elastic effective wavenumbers". In: *Waves in Random and Complex Media* 22(3), pp. 398–422. DOI: 10.1080/17455030.2012.704432. URL: http://www.tandfonline.com/doi/abs/10.1080/17455030.2012.704432 (visited on 09/04/2016).

Gower, A. (2017). *EffectiveWaves.jl: A package to calculate the effective waves travelling in materials comprised of randomly distributed particles or inclusions.* URL: https://github.com/arturgower/EffectiveWaves.jl (visited on 12/10/2017).

Kim, J.-Y. (2010). "Models for wave propagation in two-dimensional random composites: A comparative study". In: *The Journal of the Acoustical Society of America* 127(4), p. 2201. DOI: 10.1121/1.3308408. URL: http://scitation.aip.org/content/asa/journal/jasa/127/4/10.1121/1.3308408 (visited on 09/14/2016).

Linton, C. M. and P. A. Martin (2005). "Multiple scattering by random configurations of circular cylinders: Second-order corrections for the effective wavenumber". In: *The Journal of the Acoustical Society of America* 117(6), p. 3413. DOI: 10.1121/1.1904270. URL: http://scitation.aip.org/content/asa/journal/jasa/117/6/10.1121/1.1904270 (visited on 09/04/2016).

Linton, C. M. and P. A. Martin (2006). "Multiple Scattering by Multiple Spheres: A New Proof of the Lloyd–Berry Formula for the Effective Wavenumber". In: *SIAM Journal on Applied Mathematics* 66(5), pp. 1649–1668. DOI: 10.1137/050636401. URL: http://epubs.siam.org/doi/abs/10.1137/050636401 (visited on 09/14/2016).

Martin, P. A. (2011). "Multiple scattering by random configurations of circular cylinders: Reflection, transmission, and effective interface conditions". In: *The Journal of the*

Acoustical Society of America 129(4), pp. 1685–1695. DOI: 10.1121/1.3546098. URL: http://asa.scitation.org.manchester.idm.oclc.org/doi/abs/10.1121/1.3546098.

Martin, P. A. and A. Maurel (2008). “Multiple scattering by random configurations of circular cylinders: Weak scattering without closure assumptions”. In: *Wave Motion* 45(7), pp. 865–880. DOI: 10.1016/j.wavemoti.2008.03.004. URL: http://www.sciencedirect.com/science/article/pii/S0165212508000395 (visited on 12/07/2016).

Martin, P. A., A. Maurel, and W. J. Parnell (2010). “Estimating the dynamic effective mass density of random composites”. In: *The Journal of the Acoustical Society of America* 128(2), pp. 571–577. DOI: 10.1121/1.3458849. URL: http://asa.scitation.org/doi/abs/10.1121/1.3458849.

Mishchenko, M. I., L. D. Travis, and D. W. Mackowski (1996). “T-matrix computations of light scattering by nonspherical particles: A review”. In: *Journal of Quantitative Spectroscopy and Radiative Transfer*. Light Scattering by Non-Spherical Particles 55(5), pp. 535–575. DOI: 10.1016/0022-4073(96)00002-7. URL: http://www.sciencedirect.com/science/article/pii/0022407396000027.

Varadan, V. K., V. V. Varadan, and Y.-H. Pao (1978). “Multiple scattering of elastic waves by cylinders of arbitrary cross section. I. SH waves”. In: *The Journal of the Acoustical Society of America* 63(5), pp. 1310–1319. URL: http://scitation.aip.org/content/asa/journal/jasa/63/5/10.1121/1.381883 (visited on 09/14/2016).

Waterman, P. C. (1971). “Symmetry, Unitarity, and Geometry in Electromagnetic Scattering”. In: *Physical Review D* 3(4), pp. 825–839. DOI: 10.1103/PhysRevD.3.825. URL: https://link.aps.org/doi/10.1103/PhysRevD.3.825.
